# Efficacy of a mixture of neem seed oil (*Azadirachta indica*) and coconut oil (*Cocos nucifera*) for topical treatment of tungiasis. A randomized controlled, proof-of-principle study

**DOI:** 10.1371/journal.pntd.0007822

**Published:** 2019-11-22

**Authors:** Lynne Elson, Kithi Randu, Hermann Feldmeier, Ulrike Fillinger

**Affiliations:** 1 CGMRC, KEMRI-Wellcome Trust Research Programme, Kilifi, Kenya; 2 Nuffield Department of Medicine, Oxford University, Oxford, United Kingdom; 3 Dabaso Tujengane CBO, Kilifi, Kenya; 4 Institute of Microbiology and Infection Immunology, Charité University Medicine, Berlin, Germany; 5 Human Health Theme, International Centre of Insect Physiology and Ecology, Nairobi, Kenya; Hitit University, Faculty of Medicine, TURKEY

## Abstract

**Background:**

Tungiasis is a neglected tropical skin disease caused by the female sand flea (*Tunga penetrans*), which burrows into the skin causing intense pain, itching and debilitation. People in endemic countries do not have access to an effective and safe home treatment. The aim of this study was to determine the efficacy of a traditionally used and readily available mixture of neem and coconut oil for treatment of tungiasis in coastal Kenya.

**Methodology:**

Ninety-six children aged 6–14 years with at least one embedded viable flea were randomized to be treated with either a mixture of 20% neem (*Azadirachta indica*) seed oil in coconut oil (NC), or with a 0.05% potassium permanganate (KMnO_4_) foot bath. Up to two viable fleas were selected for each participant and monitored for 6 days after first treatment using a digital microscope for signs of viability and abnormal development. Acute pathology was assessed on all areas of the feet using a previously established score. Children reported pain levels and itching on a visual scale.

**Results:**

The NC was not more effective in killing embedded sand fleas within 7 days than the current standard with KMnO_4_, killing on average 40% of the embedded sand fleas six days after the initial treatment. However, the NC was superior with respect to the secondary outcomes of abnormal development and reduced pathology. There was a higher odds that fleas rapidly aged in response to NC compared to KMnO_4_ (OR 3.4, 95% CI: 1.22–9.49, p = 0.019). NC also reduced acute pathology (p<0.005), and there was a higher odds of children being pain free (OR 3.5, p = 0.001) when treated with NC.

**Conclusions:**

Whilst NC did not kill more fleas than KMnO_4_ within 7 days, secondary outcomes were better and suggest that a higher impact might have been observed at a longer observation period. Further trials are warranted to assess optimal mixtures and dosages.

**Trial registration:**

The study was approved by the Kenya Medical Research Institute (KEMRI) Scientific and Ethical Review Unit (SERU), Nairobi (Non-SSC Protocol No. 514, 1st April 2016) and approved by and registered with the Pharmacy and Poisons Board’s Expert Committee on Clinical Trials PPB/ECCT/16/05/03/2016(94), the authority mandated, by Cap 244 Laws of Kenya, to regulate clinical trials in the country. The trial was also registered with the Pan African Clinical Trial Registry (PACTR201901905832601).

## Author summary

Tungiasis is a disease caused by the female sand flea which burrows into the skin of the feet and causes intense pain and itching. People in endemic tropical areas do not have access to a simple, effective and safe method for treatment at home. The most common treatment used during clinical outreach activities is a foot bath in a disinfectant. Here we report on a clinical trial testing the effectiveness of a mixture of neem and coconut oils to kill the embedded fleas and reduce inflammation, pain and itching. We found the oil mixture reduced the proportion of fleas alive in the foot after 6 days in a similar way as the footbath in disinfectant, which is unavailable to households. However, unlike the footbath, the neem oil treatment caused most of the fleas to rapidly age in the foot and decreased the inflammation, leaving more children pain-free after one week. We conclude the neem and coconut oil mixture could be a promising approach for the treatment of tungiasis but its activity on the fleas might be slower. We need to follow up with longer observation time and test different dosages and application times.

## Introduction

Tungiasis is a neglected tropical skin disease caused by the female sand flea *Tunga penetrans* (Siphonaptera: Hectopsyllidae /Tungidae). The adult female burrows into the epidermis of its host, usually on the feet, with the final segments of her abdomen protruding above the skin. Once embedded a male will copulate with her. Males never penetrate the skin of a host but live like other flea species on the host and take blood meals. Within a week the female undergoes a remarkable hypertrophy as the eggs develop inside her abdomen, The female releases eggs into the environment through a tiny opening in the skin [1]. The eggs drop on the floor and develop in places with loose dust, soil or sand and under favourable temperature and humidity conditions, into larvae and pupae, before emerging as host-seeking adults [2] (Follow this link for Tunga penetrans Life Cycle).

The infection is associated with intense pain and itching caused by the inflammation and allergic response induced by the growing parasite and from secondary bacterial superinfections [3, 4]. In non-immunized individuals there is a risk of fatal tetanus [5, 6]. Repeated infection leads to severe pathology including the loss of nails, fissures, abscesses, ulcers and lymphedema [3, 7]. Tungiasis impairs the quality of life of children who are unable to concentrate on their lessons at school and have difficulty sleeping and walking [7–9]. Like most Neglected Tropical Diseases, tungiasis affects the most marginalized, resource-poor households, with little access to proper health care. Infection is associated with stigma and the affected are often ridiculed and consequently isolate themselves, often avoiding seeking help for fear of being disdained [10, 11].

Previous studies have attempted to identify products to treat tungiasis including oral and topical application of ivermectin, metrifonate and thiabendazole. While the oral ivermectin had no efficacy at the dose tested [12], the topical ivermectin and thiabendazole killed more fleas than the placebo lotion 7 days after treatment [13]. The use of a plant-based insect repellent containing coconut oil, jojoba and aloe vera was found to be efficacious for the prevention and control of disease if applied twice daily [14, 15]. To date, the most effective and safe method of treatment is the topical application of a two-component dimeticone (NYDA Pohl-Boskamp, Germany) [16, 17], the first-line treatment for head lice in the UK [18]. However, this product is not available in tungiasis endemic areas in sub-Saharan Africa and surgical extraction remains the main method used in affected communities. This is usually performed with sharp non-sterile instruments such as thorns, needles, safety pins and is a health hazard by itself. Moreover, consecutive use of the instrument in different individuals may lead to transmission of HBV, HCV and HIV [11]. Additionally, the rupture of the embedded flea releases eggs and *Wolbachia* bacteria into the tissues inducing an intense inflammatory response with more pain and itching [19, 20].

Tungiasis is endemic in Central and South America, the Caribbean and Sub-Saharan Africa [11, 21]. Within Kenya the disease seems to be widespread based on reports from charitable organizations [22]. According to a Ministry of Health estimate in 2013, 1.4 million people, 4% of the population had tungiasis [23]. A study carried out in 2014 of households in rural villages in Kilifi County, coastal Kenya, found the overall prevalence in the population to be 25%, and 42% of households had at least one case [24].

One treatment used widely in Kenya and recommended by the Ministry of Health for treatment of the secondary infections [23] is soaking feet in 0.05% potassium permanganate (KMnO_4_) followed by smearing with petroleum jelly. This is a very cumbersome procedure and is neither available to affected households for home treatment, nor recommended due to its severe risks of burns when handled incorrectly. Skin irritation is a common side effect [25, 26] and the purple pigmentation of the skin increases the stigmatization. In the only clinical trial using KMnO_4_ to treat tungiasis, it was found to kill less than 40% of the embedded fleas and the authors suggest the main mode of action was likely to be from the melting petroleum jelly which may penetrate the lesion and suffocate some fleas [16].

In Kwale and Kilifi counties, in the coastal region of Kenya, communities have been successfully treating tungiasis with a herbal formula comprising oil from seeds of the neem tree (*Azadirachta indica)* and coconuts (*Cocos nucifera)*[21]. Neem oil contains limonoids which are insect growth regulators, active against many arthropod species [27] [28, 29]. Aqueous or methanolic extracts of neem seeds have been reported to kill arthropod skin parasites such as *Pediculus humanus capitis*, [30], *Sarcoptes scabiei* of both dogs [31] and sheep [32, 33], and cattle ticks (*Boophilus microplus*) [34]. It is also used for the control of fleas on cats and dogs [35]. A US EPA Biopesticide Registration Document from 2012 declared “Laboratory studies indicate that the active ingredient (in cold pressed neem oil) is not toxic following oral, inhalation or dermal exposure [36].

The virgin coconut oil may also play an active role since previous studies on tungiasis have found coconut oil in combination with other plant extracts (jojoba and *aloe vera*) had a repellent affect, reducing infection and improving the condition of cases [14, 15, 37]. The fatty acid components of coconut oil have been shown to have a repellent affect against several biting flies and mosquitoes [38]. In addition, the coconut oil may well also enhance the healing process and impact secondary bacterial infection. The major free fatty acid in coconut oil, lauric acid, metabolises to monolaurin which kills bacteria by disrupting the membranes including *Staphylococcus* species, *E*.*coli* and *Pseudomonas* [39, 40]. Oleic acid increases the skin permeability allowing for better penetration of the other fatty acids in coconut oil [41], and possibly the neem oil in the trial product. In addition, coconut oil increases the speed at which wounds heal and skin repair takes place, through rapid epithelization, cross-linking of collagen, fibroblast proliferation and neovascularisation [42]. Virgin coconut oil has been shown to be efficacious for the prevention and treatment of atopic dermatitis [43] and xerosis [44].

The objective of this trial was to compare the efficacy of a mixture of neem seed oil and coconut oil with KMnO_4_ through a randomized controlled proof-of-principle study.

## Methods

### Trial design

The study was a randomized controlled proof-of-principle trial [45]. It could not be blinded to the investigators since both treatments differ significantly in appearance and application, i.e. the purple potassium permanganate solution is a footbath and stains the skin and nails for several days.

### Eligibility criteria

**Fig 1:**
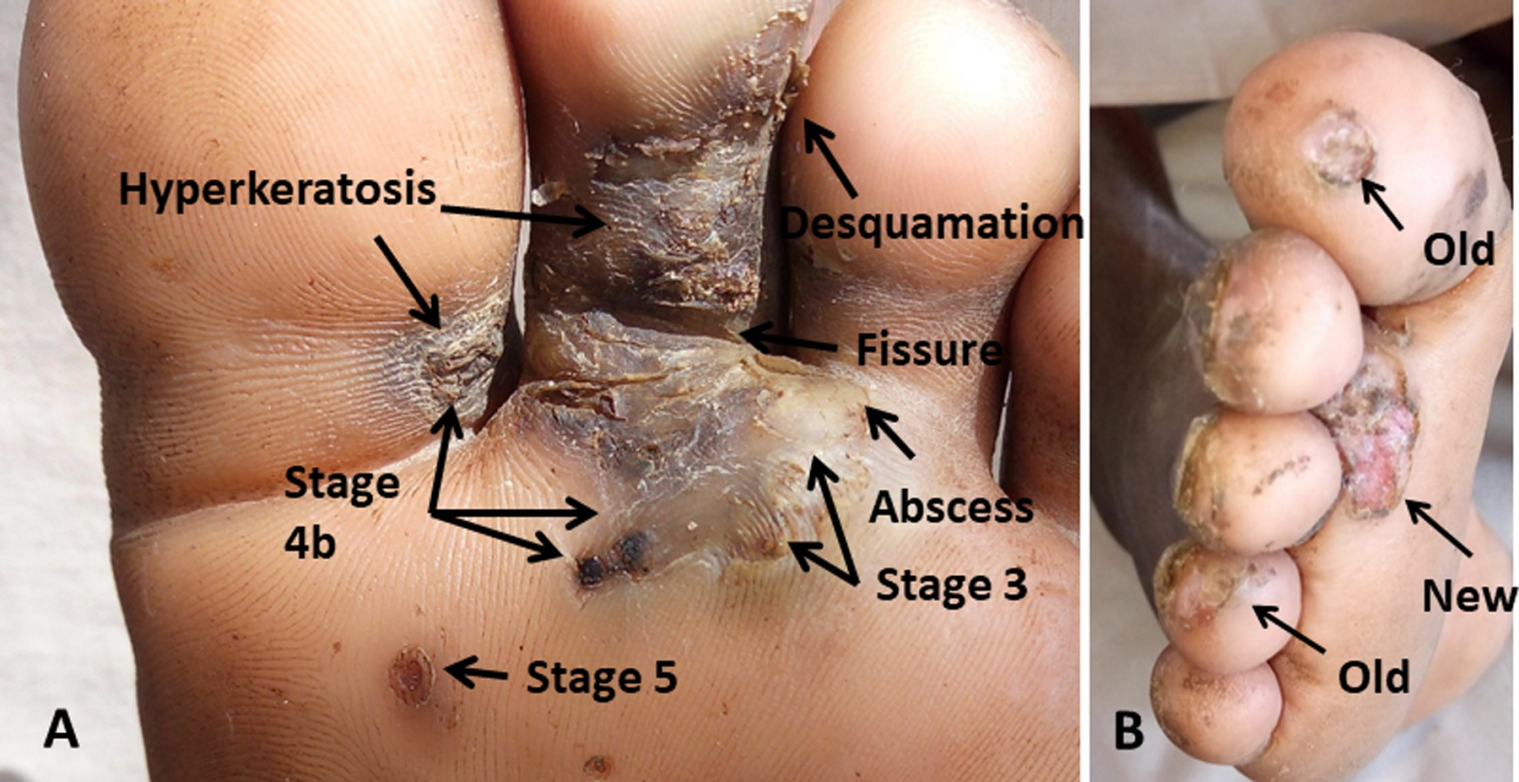
A. Fortaleza stages of embedded *Tunga penetrans* (stage 3, 4b, 5) and typical pathology (hyperkeratosis, desquamation, fissure, micro-abscess). B. Old and new manipulated lesions.

**Fig 2:**
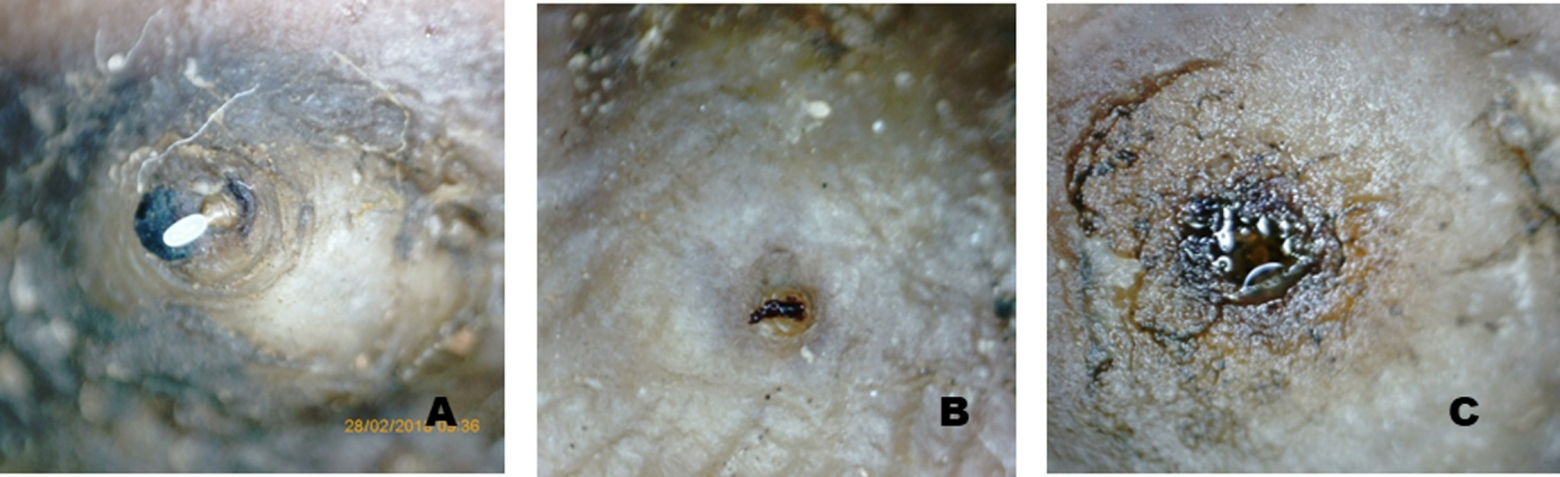
Viability signs of *Tunga penetrans*. A. Stage 3b flea expelling an egg, B. Stage 3a flea excreting a dark red/black faecal thread, C. Stage 4a flea excreting faecal liquid. Photographs taken with the digital handheld video microscope with 200x magnification.

Children aged 6–15 years were enrolled if they had at least one embedded flea of Fortaleza Stage 2-3a as originally described by Eisele et al [1] (Table 1. & Fig 1) showing at least two viability signs. Signs of viability were identified as previously defined [16]: movements of the abdominal cone, pulsations of the abdomen (S1 Video), excretion of a faecal thread, excretion of faecal fluid, and expulsion of eggs (Fig 2). Viability signs were determined by observing the embedded flea for 10 minutes using a handheld digital video microscope (DNT DigiMicro) with 3 mega-pixel optical resolution. Observations for viability with the microscope were conducted before foot washing to avoid that the washing process affected the behaviour of the fleas or washed off faecal threads and eggs. The natural development of sand fleas in the epidermis of the skin takes a period of 4–6 weeks during which the embedded sand flea passes through 4 stages of development (stage 2-4a) and eventually dies in situ (stage 4b) as described in Table 1 and illustrated in Fig 1. From stage 3a to 4a the flea releases eggs into the environment.

**Table 1:** Fortaleza stages of embedded sand fleas and characteristics (from Eisele et al 2006 [1]).

Fortaleza Stage	Characteristics
Stage 1	Active penetration of the flea into the skin. Often unnoticed by the host. Can be seen with the naked eye if observed closely.
Stage 2	Flea is completely embedded in the skin of the host and has started hypertrophy, but barely visible to the naked eye. Dark dot surrounded by erythema.
Stage 3a	Fully developed parasite with a raised yellowish-white convex surface and a central brown/black dot. Egg expulsion begins. Viability signs clear. Lasts 2–3 days.
Stage 3b	Viability signs continue to be clear, intermittent egg expulsion, rim forms around abdominal cone with concave surface. Lasts 2 weeks.
Stage 4a	Flea shrinks, becomes dry, crusted, wrinkled surface, viability signs become rare, brownish-black colouration develops.
Stage 4b	Viability signs completely absent, brownish-black colouration, abdominal structure.
Stage 5	Classic circular scar on the skin.

**Fig 3:**
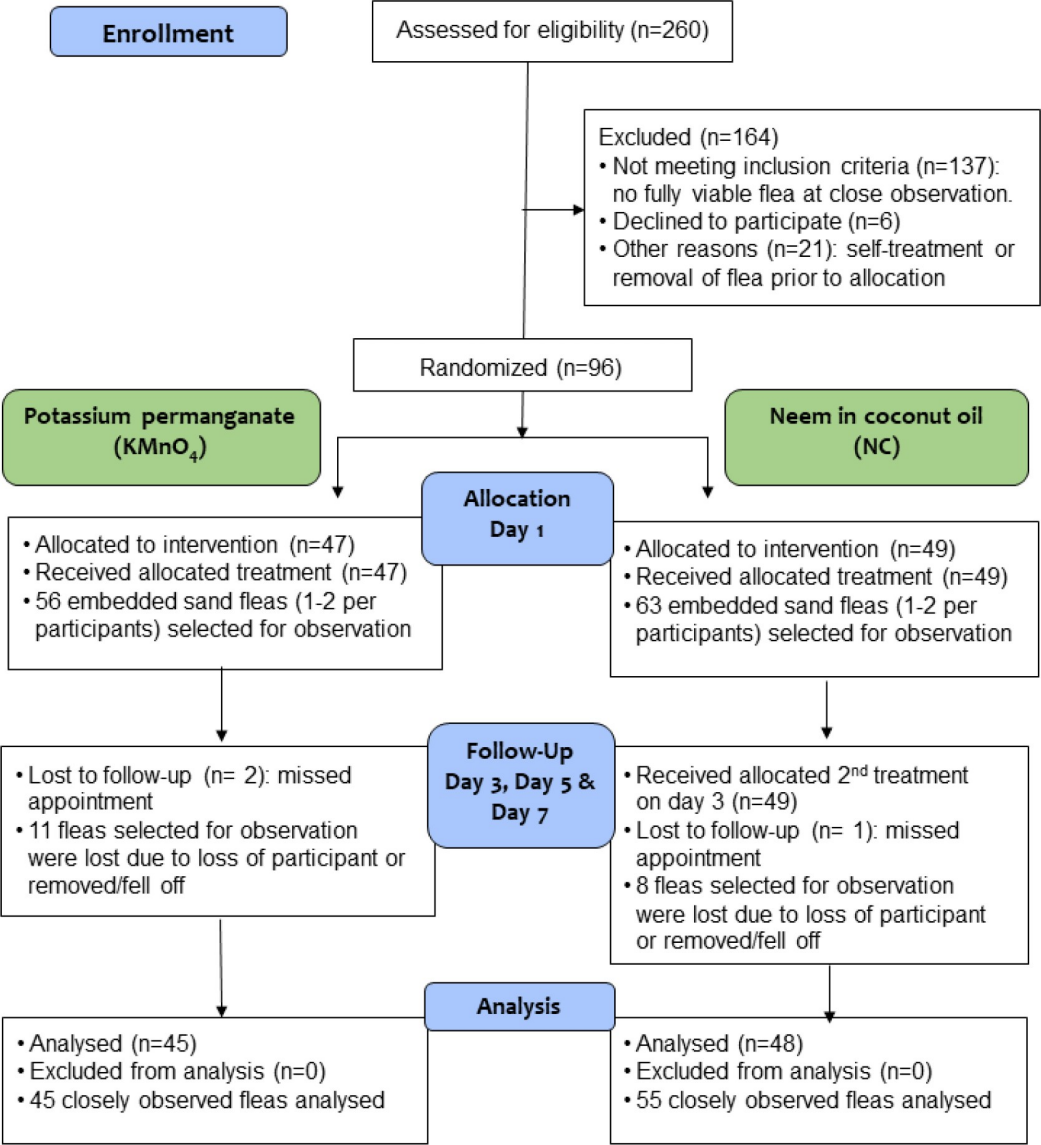
Flow chart of patients and observed sand fleas through the trial. NC = neem-coconut-oil mixture; KMnO_4_ = potassium permanganate foot bath.

Ten schools were selected based on the presence of students with tungiasis. All students in the 10 schools were screened. From these, 260 children were suspected of having tungiasis and were closely examined using the handheld microscope for viable embedded fleas. Of these, 96 had at least one fully viable flea in Fortaleza Stage 2-3a (Flow Chart, Fig 3). A maximum of two embedded, fully viable fleas were selected from each enrolled case for close monitoring to assess the impact of the treatment.

### Study area and population

The study was conducted in Kilifi County on the coast of Kenya between January and May of 2018. Primary students between the ages of 6–14 years, were included since this is the age group most affected by tungiasis [21, 24]. Children were enrolled from 10 primary schools. All but one school had stone classrooms with concrete floors, pit latrines and a water supply. Most of the population in this area lives in traditional mud-walled houses with palm thatched rooves and natural soil or sand floors. Water is obtained from shared community taps and the majority use open-defecation [24].

### Investigational product

The investigational product was a mixture of cold-pressed 20% virgin neem seed oil and 80% virgin coconut oil, henceforth referred to as NC treatment. The neem oil was produced locally in Kilifi County, coastal Kenya and sent to an ISO 9001:2015 certified laboratory prior to use for testing for potential contaminants according to WHO guidelines for assessing quality of herbal medicines [46]. There were no detectable aflatoxins, *E*. *coli*, *Salmonella*, lead, mercury, chromium and cadmium. Zinc (0.15ppm), Copper (0.11ppm), total microbial plate count (10cfu/ml), and yeast and mould counts (40cfu/ml) were all within acceptable levels according to the European Pharmacopeia [47] (S4.Annex).

At least nine neem limonoids have demonstrated an ability to block insect growth including the azadirachtin isomers and salanin [27, 48, 49]. The biochemical composition of the neem oil used in this study was analysed at the International Centre of Insect Physiology and Ecology’s Chemical Ecology Unit using gas-chromatography coupled to mass spectrometry. The total content of all azadirachtin isomers was 3.96 (95% CI 3.60–4.33) mg/ml. Salanin was the major constituent in the neem limonoids with 17.1 (95% 15.6–18.6) mg/l. The coconut oil was purchased from a commercial supplier with certification from the Kenya Bureau of Standards (no. 15501). All certificates and chromatograms are available (S5. Annex and S6. Annex).

### Treatment

Children with secondary bacterial infection were excluded from the study and referred to the nearest health facility for treatment. Eligible children were randomized to the two treatment groups using the closed envelope method. The test group was treated with NC, applied as drops of oil, using a 20 ml dropper bottle, directly to the abdominal tip of all embedded fleas. One drop of oil (approximately 0.05 ml) was applied per flea. NC treatment was done on day 1 and repeated on day 3 following community practice. Although only one or two fleas were chosen per subject for close observation of viability, all fleas found on the feet during the clinical examination were treated. The comparison group was treated, according to Ministry of Health recommendations, only once, on day 1, by placing the feet up to the ankles in a basin containing 2.5 litres of 0.05% solution of KMnO_4_ for 15 minutes followed by the application of petroleum jelly over the whole foot to alleviate the dry and rough skin caused by treatment. The children’s feet were washed thoroughly before either treatment.

Children and their parents were asked not to remove or attempt to treat any fleas during the study period.

### Outcome measures

Observations were conducted on the day of the first treatment, day 1, and then on day 3, 5 and day 7 (Fig 3).

The primary outcome measure of association in this trial was the odds ratio quantifying the impact of the different treatments on *T*. *penetrans* viability by day 7 (6 days after first treatment). An embedded flea was considered non-viable (dead) when none of the viability signs were seen during observation for 10 minutes. Videos and photographs were recorded and reviewed during the analysis phase to confirm observations recorded in the Case Report File during the study (S3. Annex).

**Fig 4:**
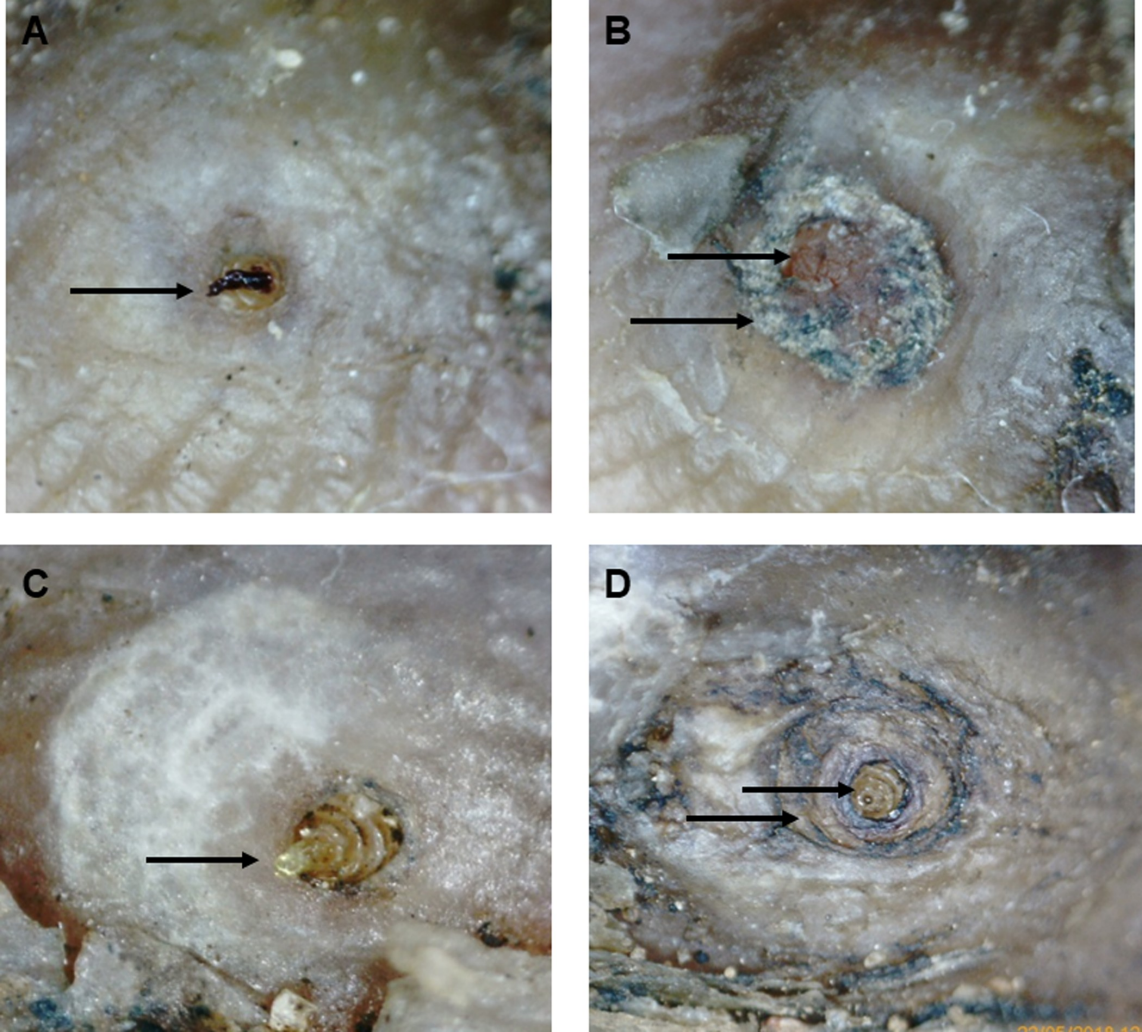
Changes in appearance of embedded fleas 7 days after treatment. Photographs taken with the digital handheld video microscope with 200x magnification. **(A)**. Viable, embedded sand flea at stage 3a with characteristic convex surface, expelling a faecal thread on Day 1 (baseline). **(B).** The same flea as in A but non-viable at day 7: loss of abdominal cone structure, surface now concave, desiccated appearance, Stage 4b.**(C)**. Viable, embedded flea stage 3a with convex surface, expelling a faecal thread on day 1 (baseline). **(D).** Rapidly Aged: same flea as in C, still viable on day 7 with clear abdominal cone, but now stage 4a, desiccated appearance, concave surface, brown colour.

Secondary outcomes included (1) the proportion of fleas with abnormal development over the 7-day observation period (unusual change in colour, shape or a 2-step change in the Fortaleza stage: ‘rapid aging’ i.e. from stage 2 to 3b, 3a to 4a or 3b to 4b; Fig 4) [16]; (2) a change in the acute pathology as measured by the severity score for acute tungiasis (SSAT) [3]; and (3) a change in experience of pain and itching subjectively assessed by the patient. The observation units for viability outcome measures were single embedded fleas, while for the SSAT, pain and itching scores the observation unit was the individual study participant.

To assess whether the treatment had an impact on tungiasis-associated clinical pathology the previously developed SSAT was used, recording the presence of erythema, warmness, oedema, desquamation, suppuration, ulcer, abscess and fissure in nine locations on both feet. In addition, the patient’s experience of itching and pain in each foot was assessed by the patient as a score in which 0 = none, 1 = mild, 2 = moderate and 3 = severe pain/itching using visual analogue scales. The SSAT has a maximum of 24 points. Since all embedded fleas were treated, it was expected that the overall clinical pathology of the feet would change after treatment.

### Adverse reactions and events

On Day 3, 5 and 7, study participants were carefully re-examined for and asked about adverse reactions such as dermatitis, fever and headache by the Clinical Officer. At each examination their temperature, blood pressure and pulse were recorded. Any adverse events were recorded in the adverse events log in the Trial Site File.

### Sample size considerations

The sample size estimation was implemented with a tool for randomized clinical trials based on the assumption that NC kills a higher proportion of fleas within 7 days than KMnO_4_ [50]. Previous reports have shown a 38% flea mortality following KMnO_4_ treatment [16] whilst estimates from community reports [21] suggested an expected 68% flea mortality following NC treatment. Hence, for sample size calculation we defined 38% as unexposed (no NC) with outcome, and 68% as exposed (NC) with outcome (flea mortality). Aiming at a balanced ratio of sample size (exposed/unexposed = 1), 80% power and significance at the 5% level, the minimal detectable odds ratio (OR) was 3.47 with 43 patients (with at least one viable embedded flea of stage 2a-3a) in each treatment arm (total 86 patients with 86 fleas). We aimed to recruit 100 patients to allow for loss at follow up.

### Statistical analyses

Each participant and each observed sand flea were assigned a unique ID to enable linkage between visits and within patients where applicable. Sand fleas were scored on day 7 as fully viable (2 or more viability signs), viable (1 viability sign), viable but unnaturally rapidly aged, or non-viable (assumed dead). Changes in pain and itching scores were analysed using generalized estimating equations with the patient ID as repeated measure and a continuous-time AR (1) correlation structure, with an ordinal logistic distribution fitted. The scores were the dependent variable and the observation day (baseline, follow up) used as explanatory variable. Generalized linear models were also used to analyse the odds of fleas being found non-viable, unnaturally rapidly aged or fully viable on day 7 as well as to analyse if an increase or decrease (binary) in pain or itching was associated with follow-up on day 7. We used generalized estimating equations to test the odds of cases reporting no pain and no itching with observation day as the explanatory variable. For this, we recorded the reported feeling of pain and itching as binary variables on day 1 and day 7 (itching: yes or no, pain: yes or no) and generated a binary variable indicating if itching and/or pain has increased or decreased on day 7 as compared to day 1 based on the reported scores. All prevalence data were modelled using binomial probability distributions with logit link functions fitted. All reported mean percentages and their 95% confidence intervals (CIs) were estimated based on the model by transforming the log odds (logit) of the outcome to the odds scale and from the odds scale to the probability scale. The Wilcoxon Signed Rank test was used to analyse the change in the SSAT from treatment day 1 to day 7 follow up. The analyses were done with R statistical software version 2.14.2 [51] and SPSS Statistics 25.

### Ethical considerations

The study was approved by the Kenya Medical Research Institute (KEMRI) Scientific and Ethical Review Unit (SERU), Nairobi (Non-SSC Protocol No. 514, 1^st^ April 2016) and approved by and registered with the Pharmacy and Poisons Board’s Expert Committee on Clinical Trials PPB/ECCT/16/05/03/2016(94), the authority mandated, by Cap 244 Laws of Kenya, to regulate clinical trials in the country. The trial was also registered with the Pan African Clinical Trial Registry (PACTR201901905832601). The study was performed in accordance with the ethical standards of the ethics committee of KEMRI-SERU, and with the Declaration of Helsinki as amended 2013 by the World Medical Association and supported by an independent trial monitor. Informed written consent was obtained from the guardians of the participants in local language (Giriama), and informed assent from the participants before data collection. The protocol is available in S1 Annex, and the Informed Consent Forms in S2 Annex. All medical examinations were implemented by a Clinical Officer. During the study, snacks were provided to the participants. At the end of the study, all participants were treated with the government recommendation of bathing the foot in KMnO_4_ and given a tetanus vaccination, provided by the nearest health facility, according to national guidelines.

## Results

Recruitment commenced 24^th^ January 2018 and ended on 16^th^ May 2018. The trial ended when a sufficient number of participants and individual fleas had been enrolled. In total 25 girls and 71 boys were enrolled. Their age ranged from 6 to 15 years, with a median age of 9 years; 47 participants (10 girls, 37 boys) received the KMnO_4_ while 49 participants (15 girls, 34 boys) received the NC investigational product. The flow chart is shown in Fig 3.

From the 96 participants, 119 embedded fleas of stage 2 or 3a were selected for close observation; in 73 participants one embedded flea and in 23 participants two embedded fleas were included. On day 7, three participants, two in the KMnO_4_ treatment group with 3 fleas and one participant in the NC group with 1 flea, were lost to follow up. In addition, a further 8 fleas were lost in the KMnO_4_ treatment group and 7 fleas in the NC treatment group. These were likely removed by the participants. Hence, included in the data analysis were 93 participants, 45 participants (45 fleas) in the KMnO_4_ and 48 participants (55 fleas) in the NC group that were treated according to protocol and examined on day 1 and day 7 (Flow chart, Fig 3).

No serious adverse events were seen during the trial. Over the observation period, three cases were suspected of having malaria and two of having chicken pox. Five cases (3 x NC, 2 x KMnO_4_) developed abscesses around the embedded fleas which is a common consequence of bacterial superinfection associated with tungiasis. Malaria and chicken pox cases were taken to the nearest facility to confirm diagnosis and treatment was obtained. All abscesses were drained by the Clinical Officer and treated with Betadine (Mundipharma Pharmaceuticals Pte Ltd, Singapore).

### Sand flea viability

At enrolment, according to the inclusion criteria, all (100%) embedded fleas selected for observation were fully viable. Significant, but similar flea mortality of 30% - 40% of all enrolled fleas was observed in both treatment arms on day 7 (Fig 4, Table 2). The decreased odds of a flea dying due to the NC treatment compared to the KMnO4 treatment was not statistically significantly (OR 0.62, p = 0.253). However, the odds of a remaining live sand flea to be fully viable (≥ 2 viability signs) on day 7 was 68% less in the NC treatment group than in the KMnO_4_ treatment group (Table 2).

**Table 2:** Treatment impact on embedded flea viability on observation day 7.

Status of embedded sand flea	Treatment	Mean %	95% CI of the mean	OR	95% CI of the OR	p value
**Non-viable**^**1**^	**KMnO**_**4**_	40	27–55	1		
	**NC**	30	20–42	0.62	0.28–1.42	0.253
**Fully viable** ^2^	**KMnO**_**4**_	41	24–60	1		
	**NC**	18	9–33	0.32	0.10–0.97	0.045

^1^ out of all observations,

^2^ out of all viable observations, excluding dead fleas

### Abnormal flea development

At baseline 6% of the fleas selected for observation over the course of the two treatments were in the Fortaleza stage 2, 82% in stage 3a and the remaining 12% in stage 3b. Whilst stage 2 and 3a naturally last for a maximum of 2 days, the embedded sand fleas remain in stage 3b for a period of 2 weeks under natural conditions. Over the observation period, most of the fleas were expected to arrive in stage 3b if unaffected by the treatments. However, we observed that among those fleas that did not lose their viability completely after treatment, a large proportion showed an unnatural fast aging process (Fig 4).

There was a significant association between the test treatment and the proportion of rapidly-aged embedded fleas. Of all those fleas that were not killed by the treatment, the odds of a flea having aged unnaturally rapidly (as compared to not having unnaturally aged) was 3.4 (95% CI 1.22–9.49; p = 0.019) times higher in the NC treatment group than in the KMnO_4_ treatment group. On average 67% (95% CI 51–80%) of all live fleas in the NC group and 37% (95% CI 21–56%) in the KMnO4 group had rapidly aged.

Dead and rapidly aged fleas are no longer reproductive. The odds were 2 times higher (95% CI 0.83–4.67)) to find a non-reproductive flea (as opposed to a reproductive flea) in the NC treatment group than it was in the KMnO_4_ group when the data for dead and rapidly-aged fleas were pooled. However, this did not reach statistical significance (p = 0.120) at the current sample size.

### Acute pathology

**Fig 5:**
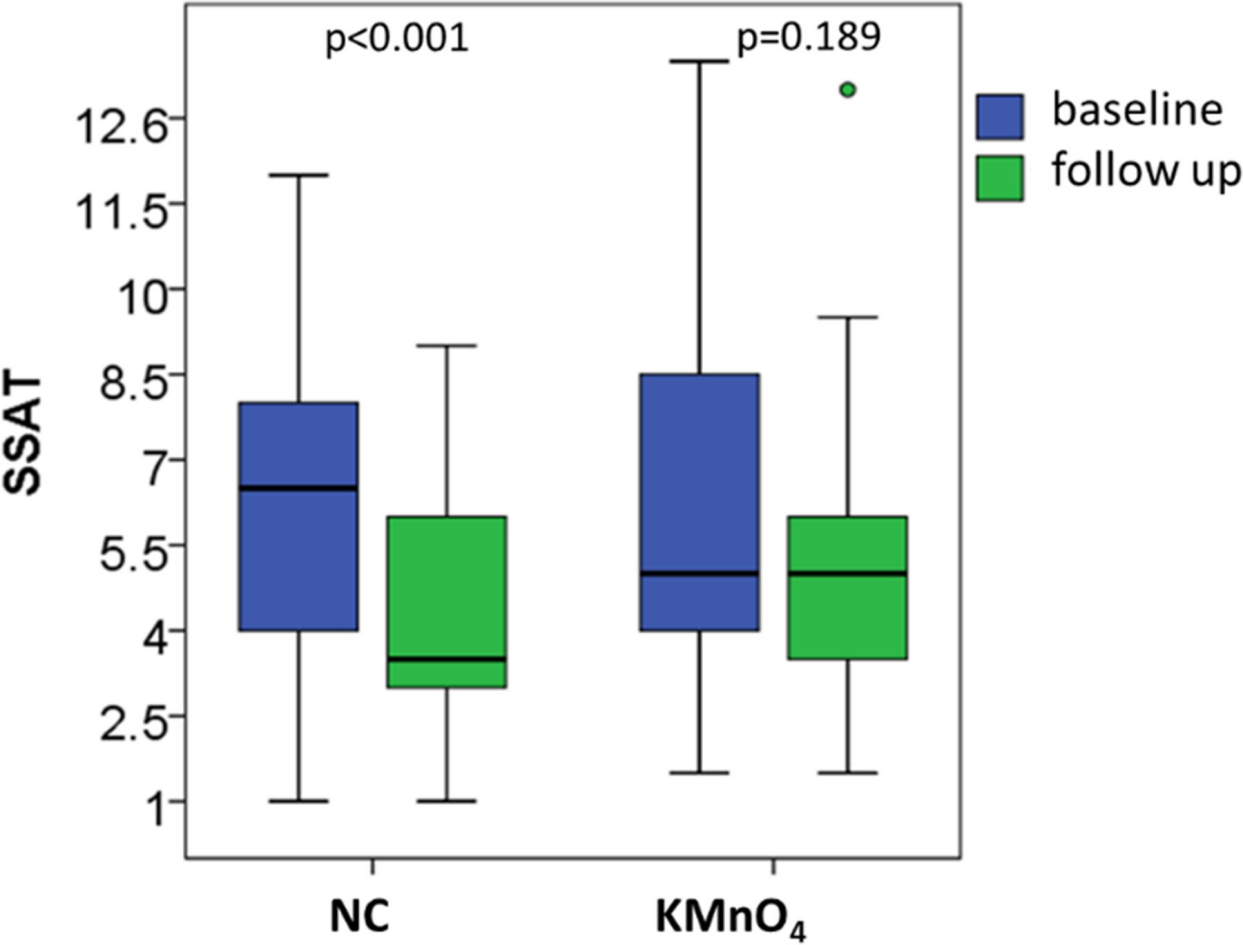
Median severity score and interquartile range for acute tungiasis (SSAT) at baseline and day 7 follow up in patients treated with NC and KMnO_4_.

For cases treated with NC, the acute pathology score, as measured by the SSAT, decreased significantly from a median score of 6.5 on day 1 to 3.5 on day 7 (p<0.001) but remained at the same median level of 5.0 for those treated with KMnO_4_ (p = 0.189, Fig 5).

### Pain and itching

**Fig 6:**
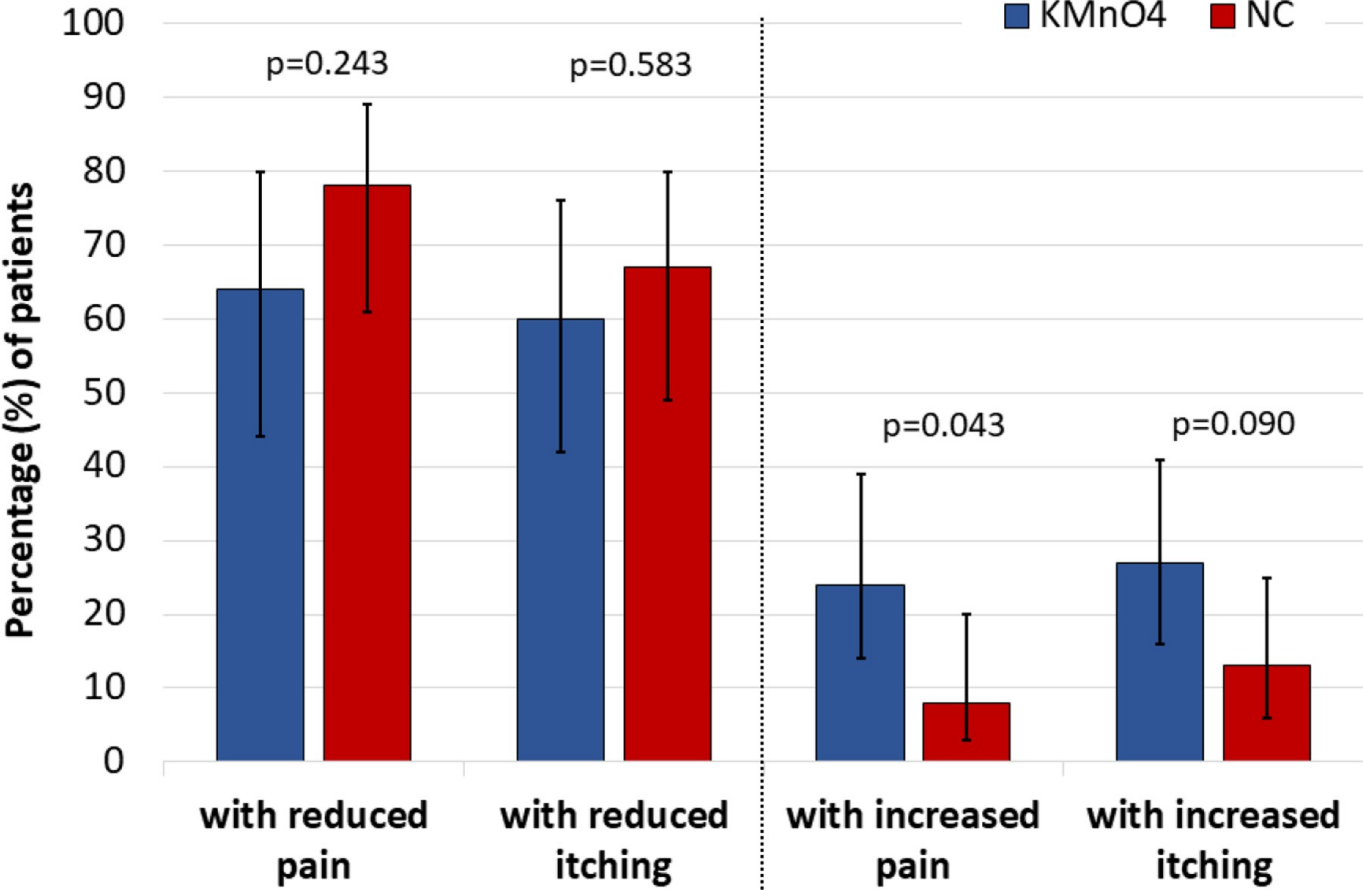
Effect of KMnO_4_ and NC treatment on tungiasis-associated pain and itching at day 7 follow up. Bars show modelled mean percentages including 95% confidence intervals. Statistical significance tested with binary logistic regression testing association between treatment and reduction/increase in pain/itching.

At baseline, 32/48 (67%) patients in the NC treatment group and 22/45 (49%) patients in the KMnO_4_ treatment group reported moderate to severe tungiasis-associated pain. Ordinal logistic regression showed a significant reduction in reported pain scores at 7-day follow up (OR 0.37 (95% CI 0.24–0.59); p<0.001) compared with baseline. This was similar for both treatment arms (OR 0.96 (0.53–1.73); p = 0.884). An estimated 64% (95% CI 44–80%) of patients in the KMnO_4_ groups and 78% (95% CI 61–89%) of patients in the NC group reported a reduction in pain (Fig 6). Similarly, a significant reduction in reported itch scores was observed at 7-day follow up (OR 0.49 (95% CI 0.31–0.77); p = 0.002); this was also similar for both treatment arms (OR 0.78 (0.46–1.34); p = 0.372). An estimated 67% (95% CI 49–80%) of the patients treated with NC and 60% (95% CI 42–76%) of patients with KMnO_4_ reported a decrease in itching at follow up (Fig 6).

In some patients, however, tungiasis-associated pain and itching increased over the observation period and this was significantly associated with the treatment. The odds ratio was 3.6 (95% CI 1.1–12.1; p = 0.043) times higher for a patient in the KMnO_4_ group to report an increase in pain, and 2.6 (95% CI 0.86–7.50; p = 0.090) times higher to report an increase in itching than for a patient treated with NC (Fig 6).

**Fig 7:**
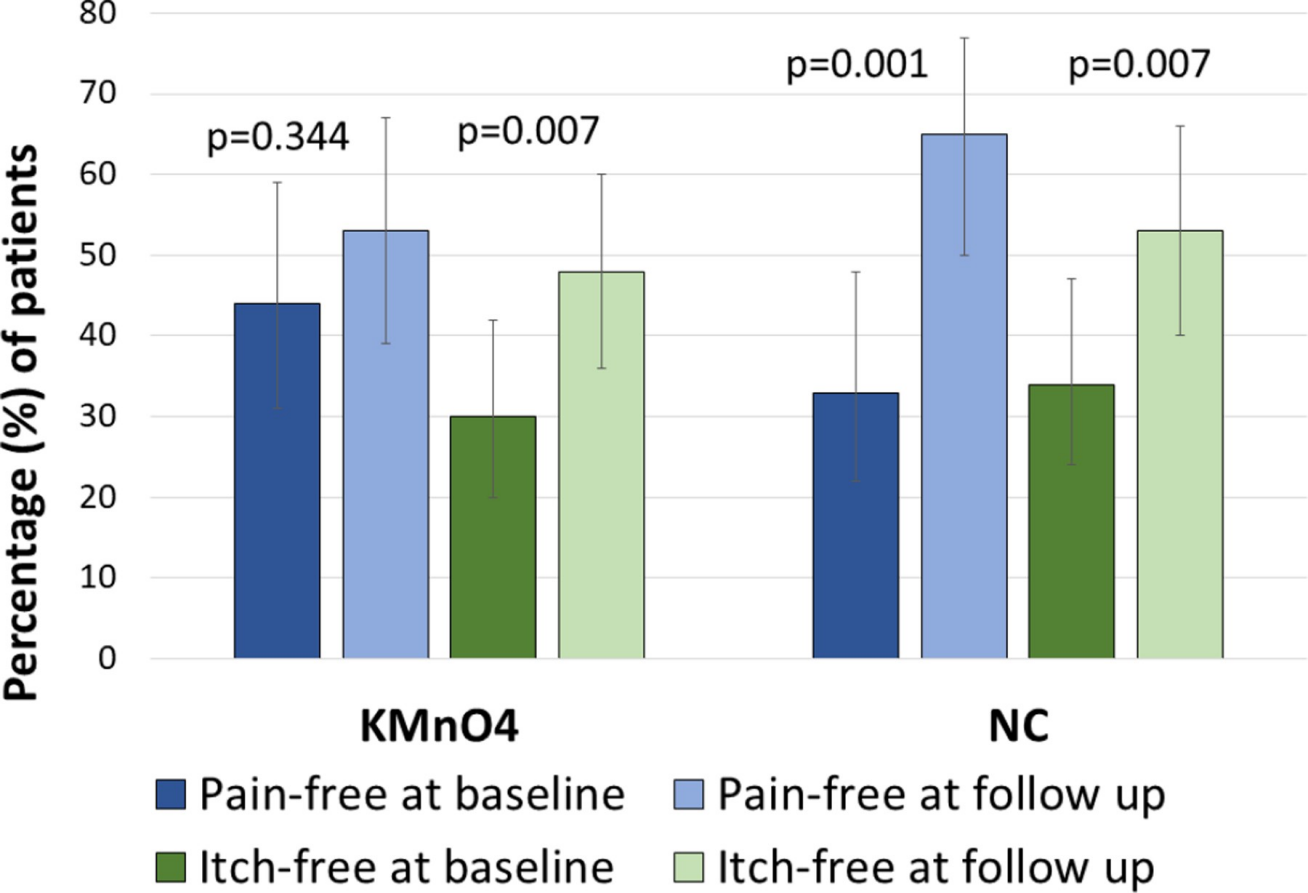
Percentage of patients being pain-free and itch-free in the KMnO_4_ and NC treatment groups at baseline and at day 7 follow up. Bars show modelled means including 95% confidence intervals.

Looking more closely at the individual experiences of pain and itching, we analysed the chance of being completely pain-free and itch-free after treatment (Fig 7). We used a binary logistic regression to test for an association between being pain-free and test treatment at baseline and 7-day follow up. A significant interaction existed between test treatment and follow up. At baseline, 44% (31–59%) of the patients in the KMnO_4_ group and 33% (22–48%) in the NC group did not have any tungiasis-associated pain. These baseline percentages for the two treatments were not significantly different. The percentage of patients that was pain-free increased slightly but insignificantly (p = 0.344) from 44% at baseline to 53% (39–67%) at day 7 follow up in the KMnO_4_ group. However, in the NC group the percentage of pain-free patients doubled from 33% at baseline to 65% (50–77%) at 7-day follow up. The odds of finding a pain-free patient (as opposed to a patient with pain) in the NC group was over 3 times higher at follow-up than at baseline (OR 3.52 (95% CI 1.70–7.28); p = 0.001). Both treatments were associated with a similar increase in itch-free patients at follow-up compared to baseline (OR 2.15 (95% CI 1.23–3.75); p = 0.007); there was no significant interaction between treatment and follow-up.

## Discussion

The neem/ coconut oil mixture used in this trial (20%: 80% v/v) was not more effective in killing embedded sand fleas within 7 days than treatment with KMnO_4_, killing on average 30–40% of the embedded sand fleas six days after the initial treatment. This efficacy matches the findings of a previous clinical trial testing KMnO_4_ [16].

However, the NC treatment led to a significant proportion of the embedded fleas to age unnaturally fast. Whilst they might have not been dead six days after the initial and three days after the second NC treatment, most of the embedded fleas had started senescence indicating imminent death. This observation is likely due to the impact of azadirachtin and other active ingredients in the neem oil which are not immediately toxic, but have a delayed impact on mortality, interfering with the normal development of insects [28, 48]. Only a fifth of the embedded fleas with viability signs remained fully viable in the NC treatment group, whilst nearly half of the fleas that had not died in the KMnO_4_ treatment group remained fully viable at day 7 follow up. Pooling the data from dead and rapidly aged fleas, suggested that the odds or finding a non-reproductive flea was 2 times higher in the NC group than in KMnO_4_ group It is highly likely that the rapidly aged fleas would have died within 24 hours after the last observation in this trial. If further studies with extended observations can confirm this estimate for NC, it would correlate well with the two-component dimeticone, which is the only highly effective treatment tested in a clinical trial and published for tungiasis control. Dimeticone has been reported to kill 78–98% of embedded fleas within 7 days [16, 17] but is unavailable in tungiasis endemic countries.

Furthermore, the current study demonstrated that the NC treatment significantly reduced the tungiasis-associated acute pathology observed by the clinician, as well as the pain and itching reported by the patients. This is likely another indicator that the fleas are dying, as it has been noted before, as the fleas lose their vital signs and eventually die, there is a concomitant reduction in acute pathology, pain and itching [17, 52]. In contrast, the acute pathology of children treated with KMnO_4_ did not decrease. Moreover, significantly more children reported an increase in pain and itching on day 7 in the KMnO_4_ group than in the NC group likely reflecting the fact that the fleas are continuing to grow.

The current study had several limitations. Budget limitations meant only a single dose of the investigational product and only a single treatment regimen, with treatments on day 1 and day 3, were tested. A higher concentration of the neem oil (i.e. 30:70% v/v) and/or one more application would have likely improved the efficacy of the product within the observation period [52]. In addition, another one or two follow up visits on day 8 and 9 would have been desirable given the slow mode of action of neem-based treatments and the high efficacy reported for NC in community-based interventions with longer observation periods [21]. Several small differences in performance of the treatments were only of borderline significance as the study was underpowered. A larger sample size would have been highly desirable to improve the power of the study but was not possible since the financial support for this trial was largely provided by well-wishers and reinforced by the commitment of a community-based group and the County Public Health Officers. Even though tungiasis has all the characteristics of a neglected tropical disease it was until very recently not included in the World Health Organisation’s (WHO) list of Neglected Tropical Diseases [53], making it difficult to seek funding from research donors. In 2017, the WHO revised its list of Neglected Tropical Diseases now including skin diseases caused by ectoparasites, hopefully paving the way for more resources to be allocated in urgently required research for tungiasis treatment and prevention. The pilot results presented here will serve as an essential baseline for planning a larger clinical trial.

Even if the topical application of NC on affected skin areas would not be more effective than the KMnO_4_ foot bath, it would still provide many advantages over the KMnO_4_ treatment being used so widely in Kenya. The absence of any adverse side effects and the ease of use for self-treatment on household level and for treatment at schools and community-based organizations that provide basic health care, provides an opportunity to reach those in need quickly and more comprehensively. We postulate that the NC induced rapid aging from a fully viable flea to a dying flea in Fortaleza stage 4a is likely to be accompanied by a rapid end of egg-expulsion. So even if the death of NC treated fleas would not be imminent, the treatment would have significant implications for breaking the life cycle and hence re-infection with tungiasis, if widely used. Further studies would enable close observation of the impact of NC on egg-laying. Additionally, the neem and coconut oils also have the bonus of including constituents which are known to be anti-inflammatory, anti-bacterial, anti-fungal and aiding skin healing [27, 40, 42, 43, 54] which likely contributed to the significant reduction in pathology in this study.

A clear advantage is the local availability of the neem and coconut trees in many disease endemic countries in sub-Saharan Africa [27], the potential for local production and relatively low costs for the product. For comparison, dimeticone, which can be purchased in Kenya only through an online pharmacy, costs Kenya Shillings (KES) 20 per ml (0.2 USD/ml). An average case will require 1ml for a single application. The costs for the tested neem-coconut oil mix, estimated based on the current local market price paid for preparing the test product, was KES 5/ml (USD 0.05/ml). An average case requires two times 1 ml for a full treatment regimen, hence costs KES 10 (USD 0.10) per patient or a maximum of KES 15 (USD 0.15) should three applications be required for optimum efficacy. It was estimated from trial expenses, that a single treatment with KMnO_4_ cost KES 34 (USD 0.34) per patient plus an additional KES 6 (USD 0.06) for Vaseline application after the foot bath (total KES 40 or USD 0.4 per patient). Hence, the treatment with KMnO_4_ is the most expensive option. In addition, KMnO_4_ is a restricted chemical due to it being an oxidizing agent, an irritant and an environmental hazard [55], it cannot be purchased by a lay person, and cannot be used for self-treatment on household or school level by non-clinically trained staff.

## Conclusion

Although 6 days after first treatment NC did not kill more fleas than the KMnO_4_, the secondary outcomes strongly suggest that if we had an additional observation day a difference would have been observed. Further trials are clearly warranted, with longer observation time and different dosages. The oils are safe, easy to handle and require only a few applications. If vulnerable families learn to apply the neem and coconut blend as soon as they are aware of a newly embedded flea, they are likely to be able to prevent it developing into a gravid female, prevent egg-laying and break transmission in their home. If provided to schools and dispensaries, it can ensure rapid and safe treatment of easily diagnosed cases as has been shown for a community-based program in coastal Kenya. There is no longer any need for families to extract the fleas, putting themselves at risk of secondary infections, nor for the continued use of KMnO_4_.

## Supporting information

S1 Annex Elson tungiasis clinical trial protocol v4-092017. (PDF)

S2 Annex Informed consent assent forms English Jan 2018. (PDF)

S3 Annex Case report forms 04012018. (PDF)

S4 Annex Contaminant certification for neem oil 05072017. (PDF)

S5 Annex Composition of neem oil 05072017. (PDF)

S6 Annex LC-Qt of-MS analysis of neem oil 05072017. (DOC)

S1 Video Movement of viable embedded *Tunga penetrans*. Stage 3a embedded flea with the abdominal tip in the centre moving and releasing a faecal thread, and pulsations of the intestines visible in the abdomen around it.
(AVI)
