# Supplementary material for: Efficacy of a mixture of neem seed oil (Azadirachta indica) and coconut oil (Cocos nucifera) for topical treatment of tungiasis. A randomized controlled, proof-of-principle study
Source: PLoS Negl Trop Dis. 2019 Nov 22;13(11):e0007822. doi: 10.1371/journal.pntd.0007822 (PMC6897421; doi:10.1371/journal.pntd.0007822)
Supplement: S1 Annex — (PDF) [file pntd.0007822.s001.pdf]

# Phase IIa, randomised controlled trial of a locally-made, herbal remedy (neem & coconut oil) for treatment of jiggers (*Tunga penetrans*).

**Protocol ID**: KEMRI-SERU Non-SSC Protocol No. 514, 1st April 2016: PPB/ECCT/16/05/03/2016(94)

**Sponsor:** International Centre of Insect Physiology and Ecology (icipe), Nairobi, Kenya. Funding for this evaluation was sought by the investigators (through *icipe*) from German Doctors, a charitable organization with the aim to support health care in low-income countries.

**Principal Investigator:** Dr. Ulrike Filinger, MSc, PhD, Public Health Entomologist, Senior Scientist, icipe- Human Health Division, Thomas Odhiambo Campus, Mbita;

**Co-Investigator/ Study Physician:** Dr. Patrick Sawa, MBChB, MSc, DTMH, Medical Officer-in-Charge, St. Jude's Clinic ICIPE, ICIPE Thomas Odhiambo Campus, Mbita

**Co-Investigator at trial site:** Dr. Lynne Elson, PhD, MPH, Public Health Specialist, Independent Consultant, Watamu. P.O. Box 770, Watamu, 80202. Mobile: 0729 338457. Email: lynne.elson@gmail.com.

**Physician at trial site:** Askah Moraa, Clinical Officer, Ministry of Health, Gede Health Centre, Kilifi County;

**Local Collaborator:** Mr. Sammy Baya, Chairman, Dabaso Tujengane CBO and WAJIMIDA Jigger Campaign, Watamu.

**Address for correspondence (PI):**

Dr. Ulrike Fillinger; Human and Animal Health Division,
International Centre for Insect Physiology and Ecology (icipe),
Duduville Campus, Nairobi 00100.
ufillinger@icipe.org, mobile: 00254-721-841452

**ABSTRACT**:

**Rationale:** Tungiasis, a parasitic skin disease (sand flea disease) inflicts pain and suffering on millions of people in Sub-Saharan Africa and South America, and yet it gets little attention from donors, scientists, governments and even health workers. There is no highly effective, widely available, safe and simple treatment. Instead communities in their desperation remove the embedded fleas using unsterilized pins and plant thorns which carry huge risk of secondary infection with bacteria and possibly even HIV and Hepatitis B. The government of Kenya currently recommends the use of potassium permanganate and Vaseline, but this has been demonstrated in a recent trial to have only 39% efficacy. Some communities use their own herbal remedies for sand flea control. In Kilifi County, a mix of natural products based mostly on neem oil (*Azadirachta indica*),*)* and coconut oil (*Cocos nucifera*) known as JIGFIX, is used by local community self-help groups. Coconut oil is widely used in the food and cosmetics industry and neem oil as an antiseptic, and used for medical and agricultural insect control. Their safety and beneficial properties have been shown in various contexts. Their combined effect on pathology, bacteria, fungi or insects has never been tested scientifically.

**Primary Objective:** To determine whether a mix of neem and coconut oils kills a higher proportion of embedded jiggers than the current standard of potassium permanganate and Vaseline, in 7 days.

**Secondary Objectives:**

- To determine whether neem and coconut oil reduces inflammation, pain and itching better than the standard treatment, in 7 days.
- To determine the safety of the neem and coconut oil mix for topical use.

**Methods:** The study will follow a randomized controlled trial design. Up to one hundred school children aged 6-14 years with at least one clear live, embedded flea at stage II and III will be enrolled. They will be randomized to be treated with either the study product, the neem and coconut oil mix or with potassium permanganate. The study will take place in Kilifi County. The embedded fleas of each participant will be monitored every other day for 7 days after treatment using a digital handheld microscope for signs of viability, abnormal development and inflammation. The children will also be asked to assess their level of pain and itching, the major symptoms caused by embedded fleas.

**Application of Results:** If the neem and coconut oil mix proves to be more effective at killing embedded fleas than the current standard of potassium permanganate, this study will have huge implications, not only for Kenya, but also for all countries where jiggers are endemic. The neem and coconut oil mix will be the only effective, safe and simple treatment available.

## Contents

2. Background Information ....... 5
Tungiasis ....... 5
Treatment options ....... 6
Investigational Product ....... 7
2.a. Efficacy of Neem ....... 7
2.b. Risks and benefits to human subjects ....... 8
2.c. Trial Product Dosage ....... 12
2.d. Statement ....... 12
2.e. Study Subjects ....... 13
3. Trial Objectives and Purpose ....... 13
4. Trial Design ....... 13
4.1. Study Design ....... 13
4.2. Primary and secondary endpoints ....... 15
4.3. Measures to minimize/avoid bias ....... 15
4.4. Duration of subject participation ....... 16
4.5. Discontinuation criteria ....... 16
4.6. Accountability procedures ....... 16
4.7. Maintenance of trial treatment randomization codes ....... 17
4.8. The identification of any source data. ....... 17
5. Selection and withdrawal of study participants ....... 17
Inclusion criteria ....... 17
Exclusion Criteria: ....... 18
Withdrawal criteria ....... 19
6. Treatment of study participants ....... 19
The Investigational Product ....... 20
Participant's compliance ....... 21
7. Assessment of Efficacy ....... 21

Primary Outcome: ... 21
Secondary Outcome: ... 22
8. Assessment of Safety ... 22
Adverse Events/Serious Adverse Events ... 22
Adverse events treatment ... 24
Adverse events facilities ... 24
9. Statistics ... 24
9.1 DSMB ... 24
9.2 Sample Size ... 25
9.3. Sampling procedures ... 25
9.4. Randomization Process ... 26
9.5. Data Management ... 26
10. Direct Access to Source Data/Documents ... 27
11. Quality Control and Quality Assurance ... 28
11.1. The Investigators ... 28
11.2. Monitors and monitoring plan ... 29
12. Ethics ... 30
12.2. Treatment and/or management after completion of trial ... 32
12.3. Confidentiality ... 32
12.4. Follow-up of trial study participants ... 32
12.5. Insurance and indemnity measures ... 32
13. Data Handling and Record Keeping ... 33
Data Storage ... 33
14. Publication Policy ... 34
15. Bibliography ... 35

## 2. Background Information

### Tungiasis

Tungiasis is a tropical skin disease caused by the female sand flea *Tunga penetrans.* In East Africa it is known as jiggers, in Kenya as funza. The adult female burrows into the epidermis of its host, usually on the feet, with the final segments of her abdomen protruding. The female is fertilised by a male whilst the female is already embedded in the skin (1). Both sexes suck blood. The female undergoes massive growth as the eggs develop inside her abdomen and are released out into the environment where they develop in dry soil into larvae and pupae, before emerging as infective adults themselves (1). Tungiasis is a zoonosis. Domestic animals such as dogs, cats, goats and pigs, and sylvatic animals such as rats, have been found to be similarly infected by *Tunga penetrans* and serve as reservoirs (2) (3) (4).

Tungiasis is one of the most neglected tropical diseases, reaching epidemic proportions among resource poor and rural communities in Sub-Saharan Africa, South America and the Caribbean (5). In Kenya alone the Ministry of Health estimate two million people are currently infected. A survey across Malindi sub-county of Kilifi County in November & December 2013, found an overall prevalence of infection in adults and children of 15% (MOH Malindi, unpublished). The majority (75%) of those infected are children under 15 years of age, plus the elderly (Elson, 2013 unpublished). Less mobile elderly and disabled people as well as alcoholics may present severe morbidity with hundreds of penetrated sand fleas (6) (7).

The infection is associated with intense itching and pain caused directly by the growing parasite and also from secondary infections with bacteria (8). In non-immunized individuals there is also the risk of fatal tetanus infection (9) (10). Children are unable to concentrate on their lessons at school and have difficulty sleeping and walking (5) (8) (9) (11). Infection is associated with stigma. Victims are often ridiculed and consequently isolate themselves and often avoid treatment for fear of being disdained. Since tungiasis infestation hinders walking and concentration, it impacts children's ability to learn and in future to get jobs. Tungiasis, affects adults' ability to conduct their work, whether it be a paid job or in their own farm. Thus, there is a vicious cycle between poverty exposing

people to tungiasis and the infestation causing more poverty and ultimately impacting the socioeconomic status of the population.

### Treatment options

Until recently, there were no effective, safe methods of chemotherapy and extraction was the main method used by affected communities. However, this is extremely unsafe, since it is conducted with thorns or other locally available sharp implements without soap, water or other disinfectants, resulting in a high risk of bacterial infection. Physical injury and haemorrhage will also be caused by the extraction and can lead to transmission of HIV and Hepatitis B virus when the instruments are used consecutively for multiple cases (as is common). Additionally, rupture of the embedded flea releases eggs and endosymbiotic bacteria into the tissues inducing a greater inflammatory response with more pain and itching (12) (13)

Until now, the only treatment recommended by the Ministry of Health in Kenya has been a 15 minute soak of the feet in 0.05% potassium permanganate baths followed by drying and covering with petroleum jelly. This is not only a very cumbersome procedure, but a recent clinical trial in Kenya showed that it is only marginally effective, with only 39% of embedded fleas showing signs of non-viability, 7 days after treatment (14) . The potassium permanganate treatment may also increase the stigma, since it turns the skin purple, making treatment visible to others.

A novel method of treating tungiasis has been trialled in Madagascar and Kenya, NYDA®, a two-component dimeticone (silicone oils) with low viscosity and high creeping properties used widely in the shampoo industry and as treatment for head lice in Europe, USA and Canada (15) proved to be highly effective, with 78% of embedded sand fleas losing viability and inflammation reducing 7 days after a single application. However, NYDA® has a few challenges to its widespread use. Firstly it is flammable and therefore treated patients must stay away from open fires. Secondly NYDA® is expensive and currently not available in Kenya.

Previously, studies in Brazil demonstrated that a product sold in Germany as a natural insect repellent, Zanzarin, containing a mix of coconut oil, jojoba and aloe vera was effective

against tungiasis if applied daily (16). Unfortunately, this product is no longer being manufactured. Recently in Kenya a study demonstrated that coconut oil alone will work as a repellent, reducing infection and acute tungiasis symptoms, but only if applied twice daily for a month (17). Communities of Kwale and Kilifi counties of Kenya have been successfully treating their own jigger infections with coconut oil alone or a herbal medicine known as JIGFIX (Turihabz Investments, Mombasa). Its main components are neem oil (*Azadirachta indica)* and coconut oil (*Cocos nucifera)*.

### Justification

The Kenya Ministry of Health developed in 2014 the first 'Kenya Policy Guidelines on Jigger Prevention and Control, 2014' and this policy document highlights very well the knowledge gaps and urgent need for operational research. The document concludes as follows: 'Research on jigger prevention and control needs to be prioritized because literature on the subject is scanty and there is need for additional authoritative knowledge on jiggers, their prevention and control. However, such research needs to be carried out in collaboration with academic institutions, with approval by the health ministry's ethics committee, for more rigour and credibility.' It is in this context that this study aims to compare the efficacy of a neem and coconut oil mix, to the government recommended standard, potassium permanganate and Vaseline, to investigate if the locally produced, low-cost treatment would provide a sustainable and superior alternative to current practice.

## Investigational Product

The investigational product is a simple mix of 20% neem oil in virgin coconut oil.

## 2.a. Efficacy of Neem

Neem oil might be expected to be effective against *Tunga penetrans* since neem has been used for thousands of years to control insects on crops and in food storage (18). Scientific studies have demonstrated neem extracts to be a powerful insect growth regulator, a feeding deterrent and repellent with low toxicity. It has been shown to control hundreds of arthropod species including agricultural pests and is used widely in organic farming as a natural pesticide (18). In addition, neem oil has been reported to kill arthropod skin parasites such as

head lice (*Pediculus humanus capitis*, (19)), scabies *(Sarcoptes*) of both dogs (20) and sheep (21) (22), and cattle ticks (*Boophilus microplus*) (23) as well as for the control of fleas on cats and dogs (24). Spraying of neem extracts on water has also been shown to kill mosquito larvae (25) (26) (27) and is in some areas used for malaria control. Neem oil is frequently used for preparing cosmetics (soap, hair products, body hygiene creams, hand creams).

Both the neem and coconut oils have been demonstrated to have antibacterial, antifungal and anti-inflammatory effects in controlled trials and have been used for such, for thousands of years in India (28). Inflammation is the leading pathology associated with embedded fleas. Secondary infections with bacteria are extremely common, exasperating the pain experienced. Embedded fleas commonly have commensal *Wolbachia* bacteria which are released if the fleas are cut open inducing a massive inflammatory response (13). Often patients also have fungal infections between the toes.

The neem and coconut oil mix has never been tested scientifically so its efficacy and the exact mode of action is unknown, but it could be working on several levels at once; killing the embedded flea as well as the bacteria and reducing pathology.

## 2.b. Risks and benefits to human subjects

### Toxicology and toxicity of the neem and coconut oil mix ingredients

Since coconut oil is a highly nutritious food source used globally and is used widely in the cosmetics industry, it is expected that the only component that may have any toxicity is the neem oil. There is no data available on the toxicity of the combination of the 20% neem and coconut oil that we will use. However, a review of trials in humans for insect repellency reported 5% neem oil in coconut oil to be “Non-irritating to skin and mucous membranes” (29). In addition, a 20% methanolic extract of neem seed kernals in Vaseline caused no skin irritation or restlessness among sheep treated for Sarcoptes scabiei when applied 7 times over 14 days (22).

### Neem oil

U.S. EPA TOXICITY CATEGORY: IV for oral exposure: “low toxicity”.

Acute toxicity testing has been conducted for whole neem oil on rats and rabbits and the $LD_{50}$ calculated to be 14 and 24ml/kg bw/day respectively (30). This would equate to oral ingestion of 420ml-720ml of neem oil by a child of 30kg (10 year old), or 210-360ml for a child of 15kg (4 year old). The toxic effects observed included motor activity, respiration, diarrhoea, tremors and convulsions. The most significant adverse effect was considered to be on the fertility of adult females from sub-acute exposure at 2.0-4.6 mg/kg bw, which was reversed once neem oil intake was discontinued. However, another study in Germany using neem oil extracted from clean seeds found even doses as high as 5g/kg induced no signs of toxicity in rats (31), and others reported no effect on biochemistry or histopathology after 90 days of daily oral exposure at does as high as 1.6g/kg (32) (33)

There have been reports of acute toxicity to humans. One child experienced toxic encephalopathy, vomiting, drowsiness, tachypnoea, and recurrent generalised seizures after ingestion of 5ml of neem oil, but recovered (34). Another child was reported to have died as a result of ingestion of 12ml of neem oil over 2 days, but the case report also considered the possibility that the effect was due to aflatoxin in the neem preparation (35).

Based on the reversible fertility effect, the human estimated safe dose (ESD) for oral exposure to unprocessed oil is 0.26mg/kg bw/day (30) and the Acceptable Daily Intake (ADI) of neem oil to be 0.14ml of oil/day for an adult (0.06ml for a child of 30kg) if taken orally (30).

It should be noted that these assays and safe dosages are for oral administration, while the neem and coconut oil mix in this study will only be used topically. The US EPA classifies cold pressed neem oil as Category III for dermal exposure, "slightly toxic" and the US National Pesticide Information Center reports that it can be slightly irritating to eyes and skin (36). A factsheet of the US EPA for Cold Pressed Neem Oil (025006) states: "No risk to human health is expected from the use of Cold Pressed Neem Oil because of its low toxicity via all route of exposure. Cold Pressed Neem Oil has been used for hundreds of years to control plant insects and diseases. EPA concluded that Cold Pressed Neem Oil is not a mutagen, and is not a developmental toxicant. Based on the review and analysis of the guideline studies, no additional toxicity data are required to support food uses of this biochemical" (37).

In this study each study subject will receive 0.05ml to 1ml of the neem and coconut oil mix directly on the embedded flea, on two days. Since Neem oil is 20% of the test product, the subjects will be exposed to a maximum of 0.2ml of neem oil in a dose, twice. For an average child of 30 kg this converts to 6.7ug/kg of neem oil, twice, 39 times lower than the Estimated Safe Dose and 298 times lower than the dose which caused reversible fertility effects in rats (Table ).

**Table: Toxicity of neem oil**

| **Indicator** | **Dose quoted** | **Dose equivalent** | **Relative to dose to be applied dermally (1ml/day= 6.7ug neem/kg)** |
|---|---|---|---|
| LD50 rats oral | 14ml/kg | 14mg/kg | <2,089x |
| LD50 rabbits oral | 24ml/kg | 24mg/kg | <3,582x |
| Fertility Effects rats oral | 2mg/kg | | <298 |
| Sub-chronic 90 days LD50 rabbits oral | 5000mg/kg | EPA | |
| EPA category for oral | IV | Not toxic | |
| EPA category for dermal irritation | III | Not dermal sensitizer | |
| Estimated Safe Dose | 0.26mg/kg | | <39x |

**Nature and degree of risk associated with participation in the study**

| | **Nature of Risk** | **Degree of Risk** |
|---|---|---|
| 1 | Adverse reactions to the neem and coconut oil mix. | Unlikely. Neem and coconut oil has been used widely in Kilifi and Kwale Counties with no reported adverse reactions to it. (Dabaso Tujengane CBO, Prof. Karama, KEMRI, personal communication). Furthermore, neem oil has been extensively tested for other medical applications and for use in the cosmetics industry. Only a small amount will be used and only for **topical** application. The application will be done under medical supervision. |
| 2 | Adverse reactions to potassium permanganate/ Vaseline | Unlikely. |
| 3 | Stigma & discrimination. Subjects | Likely, as this is usual practise in the community. |

| | | |
|---|---|---|
| | face the risk of being ridiculed publicly by their peers for having jiggers. | All subjects will be seen to be selected for the jigger study, will have one foot stained purple by the permanganate, and therefore identified as having jiggers. |
| 4 | Missing lesson time. Children will be removed from their lessons for at least 30 minutes on 3 days of one week. | Highly likely. This time out of class is considered to be of "minimal risk" since it is not any more lesson time missed than children normally experience in any given year as a result of being sent home for lack of levies etc. In addition, we anticipate that the amount of lesson time the children will miss will not have any more impact on the child's learning than being present in the class scratching and being in misery from the active jiggers. |

**Minimization of risk**

| | **Nature of Risk** | **Minimization of Risk** |
|---|---|---|
| 1 | Adverse reactions to the neem and coconut oil mix. | No more oil will be used than directed by the manufacturer. The Clinical Officer will examine all subjects for adverse events and will prescribe suitable treatment and referral if necessary. |
| 2 | Adverse reactions to potassium permanganate/ Vaseline | National guidelines will be followed. The Clinical Officer will examine all subjects for adverse events and will prescribe suitable treatment and referral if necessary. |
| 3 | Stigma & discrimination. Subjects face the risk of being ridiculed publicly by their peers for having jiggers. | Before the study commences the investigators will give a talk to all students and staff to introduce the study, explain jigger infection and prevention and the importance of not stigmatizing affected children. |
| 4 | Missing lesson time. Children will be removed from their lessons for at least 1 hour on 3 days of the week. | The treatment will actually enable the children to concentrate and benefit from the subsequent lessons. |

**Benefits**

**Subjects**

The individual study subjects will benefit from the study immediately by receiving treatment of their jiggers. For everyone in the school, they will receive screening for jiggers and soon after the end of the study will be treated with the most effective intervention. For those children with abscesses associated with their jigger infection, they will not be enrolled in the study but transport will be provided to the nearest health facility for simple, single course antibiotics and analgesics.
For all children in the school there is the opportunity of seeing the clinical officer. If found suspected to have any other diseases, they will be referred to the nearest clinic. They will not be provided with transport and the study will not pay for their treatment for non-jigger related diseases.

**Society**

The findings of this research study will have immense impact on society in general if indeed the the neem and coconut oil mix is found to be more effective than the current standard. It will mean availability of an effective, safe, convenient, local and affordable treatment option. Communities will no longer need to take the risk of injury and secondary infection from non-sterile thorns and blades.

## 2.c. Trial Product Dosage

*Tunga penetrans* is an ectoparasite, embedded in the epidermis of the feet, particularly the peri-ungal areas. The embedded flea always maintains an opening to the outside through which it respires and excretes waste. Thus, the most effective route of administration of any treatment is topical, directly onto the embedded flea.

The original herbal medicine, known as JIGFIX, is usually applied only twice to the embedded fleas. Thus for this first 'Proof of Principal' study for the neem and coconut oil trial product, we chose the same dosage and dosage regimen. The oil will be applied, one drop (approximately 0.05ml**)** directly onto the embedded flea and spread over the immediately surrounding inflamed skin on 2 days.

## 2.d. Statement

The trial will be conducted in compliance with this protocol, GCP, National and PPB requirements.

### 2.e. Study Subjects

The study will recruit 100 subjects from the appropriate classes of a public primary school. These will include children, both boys and girls, aged 6 to 14 years. Less than 100 subjects may be enrolled if some subjects have 2 eligible embedded fleas which can be clearly monitored. The ceiling will be 100 embedded fleas on a minimum of 50 subjects.

Children are considered **'persons of limited capacity for autonomy'** and will be part of the study since they comprise the majority of the victims of jiggers and are the main intended beneficiary population.

## 3. Trial Objectives and Purpose

**Primary Objective:** To determine whether the neem and coconut oil mix kills a higher proportion of embedded jiggers than the current standard of potassium permanganate and Vaseline, in 7 days.

**Secondary Objectives:**

i. To determine whether the neem and coconut oil mix reduces inflammation, pain and itching better than the standard treatment, in 7 days.
ii. To determine the safety of the neem and coconut oil mix for topical use.

## 4. Trial Design

### 4.1. Study Design

The study will be conducted as a Phase IIa randomized, controlled trial, i.e. a Proof of Principle and safety study.

The study will include children aged 6-14 years who have viable embedded sand fleas in both feet. This will be assessed by handheld digital microscope for expulsion of eggs, excretion of faecal thread, excretion of faecal liquid, pulsations/contractions.

Each study subject will be randomized to one of the two treatment arms, the embedded fleas will be treated with the neem and coconut oil mix or with potassium permanganate, the national standard (National Policy Guidelines for Prevention and Control of Tungiasis 2014[1]) and most widely used treatment in the country, although not registered for the treatment of tungiasis. Potassium permanganate solution is used in dermatological practice for various skin conditions, e.g for treatment of fungal infections.

**Figure 1. Flow chart of study design**

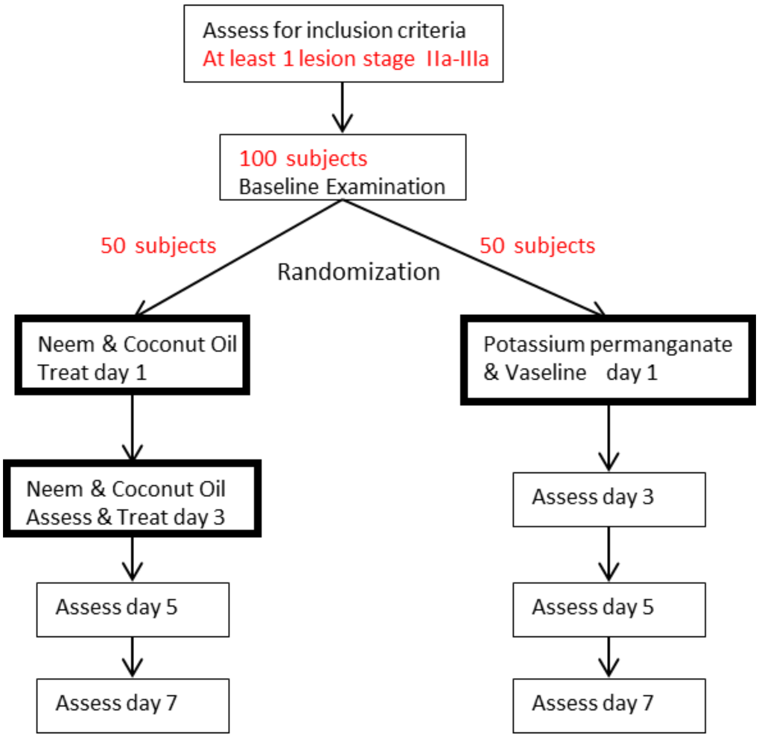


[1] available at http://www.jigger-ahadi.org/National%20Policy%20Guidelines%20for%20Prevention%20and%20Control.pdf

The assessments on Day 3, 5 and 7 will each include:

- Observations of feet for clinical symptoms: inflammation, ulcers, suppuration
- Signs of adverse effects of the product such as dermatitis
- Individual embedded fleas selected for the study will be observed for 15 minutes for viability signs using a digital handheld microscope.
- Subjects experience of pain and itching as assessed using Visual Analog Scales (Annex 3)

## 4.2. Primary and secondary endpoints

For the primary objective, the unit of observation are individual flea lesions located on the right or the left foot and the Primary Endpoint will be the loss of signs of viability of the individual flea (expulsion of eggs, excretion of faecal thread, excretion of faecal liquid, pulsations/contractions).

For the secondary objective the unit of observation will be the individual study subjects and the Endpoint will be the end of the study at Day 7.

## 4.3. Measures to minimize/avoid bias.

Study subjects will be assigned to the study product or the control treatment groups on enrolment using simple randomization. The first subject will pick a card from an envelope containing 2 cards, 1 with “neem” written on it, the other with “permanganate” written on it. The selected card will be the treatment received. To ensure equal numbers of subjects are treated with each product, the subsequent subjects will then alternate between the 2 products. The study cannot be blinded as the products are different in appearance and application and the permanganate stains the skin for several days.

There will be no placebo control group as it is not ethical to not treat any children found to have jiggers since effective treatments are available and the infection is so debilitating.

## 4.4. Duration of subject participation

Due to the time needed for observation of each of the selected lesions (15 minutes) every second day, the subjects will be divided into groups. A maximum of 14 subjects can be examined in a day by one investigator However, since every lesion must be examined every other day for 7 days, only 2 groups can be enrolled in one week (staggered by a day). Once their observations are complete a third and fourth group will be started in the second week. This is illustrated in Annex 1. If less than 14 subjects are available on enrolment days, the study will continue for more weeks until the total 100 subjects are enrolled and observations are complete.

Data entry and analyses are expected to take another 1 month after the completion of the field trials.

## 4.5. Discontinuation criteria

Since this trial only involves 2 applications of the trial product, will only take two weeks to complete, the participation of each subject will only be 7 days and there is a very small likelihood of adverse events from the trial product, it is not expected that any subject or the trial will have to be discontinued. However, should any individual subject be determined to have adverse effects as a result of the trial product they will not receive the second dose and will be withdrawn from the study. The entire study will be discontinued if 10% of the study subjects experience adverse effects from the trial product.

If a subject removes the embedded flea, or applies any other product to the flea they will be eliminated from the study.

## 4.6. Accountability procedures

The study will only require a total of 300ml of the trial product and will be produced by a qualified laboratory technician at icipe using source oils purchased from a single batch. The

control product, potassium permanganate and Vaseline will be obtained from the Department of Health in Kilifi County, as a single batch.

Records will be kept of the purchase of all items.

## 4.7. Maintenance of trial treatment randomization codes

There are no randomization or blinding codes in this study.

## 4.8. The identification of any source data.

Records for the research project will be stored in a secure location (locked filing cabinets in locked room). Study information containing personal identifiers stored on computers and other storage devices will be password protected to prevent unintentional breaches of confidentiality in the event the storage device is lost or stolen. Similarly, paper records identifying research subjects including consent forms will be kept under the personal control of the core researchers. Consent forms and paper data records will be locked in file cabinets in offices at the performance site and at icipe-Nairobi. Electronic Data Records will be stored in password protected files, on icipe maintained servers with regular back-up. Research data will be retained for 3 years after the completion of the project. Records may be preserved in hard-copy, electronic or other media form, and will be accessible for audit purposes. Records for completed projects will be stored in secure locations at icipe with the same care used when the project was active.

# 5. Selection and withdrawal of study participants

## Inclusion criteria

- Children age 6-14 years, both male and female.

This age group is targeted as the age most affected by jiggers and also able to give assent for participation in the study.

- With one or two viable, embedded sand flea of stage IIa-IIIa of the Fortaleza classification, on both feet.

In IIa the sand flea is already completely embedded in the skin of the host and has started to grow (hypertrophy). Lesions in stage IIIa correspond to a fully developed parasite with a characteristic watchglass-like appearance. In this stage the female sand flea starts to expel eggs. In stage IIIb egg expulsion stops, thereafter the sand flea dies and the lesion changes into stage IV. Thus, sand fleas in stage IIa – IIIa are most suitable to assess viability and alterations in the normal development of the parasites.

The inclusion criterion for an eligible lesion will be the presence of at least 2 out of 3 viability signs at the baseline examination: expulsion of eggs, excretion of a liquid faeces or faecal threads, pulsations/ contractions of the parasite. Viability signs will be determined using a handheld digital video microscope (portable dnt DigiMicro Mobile).

- Availability of a caregiver over the age of 18 years to sign the consent forms. Caregivers of selected children will be requested to attend school to sign the consent forms.

## Exclusion Criteria:

Children with too few or too many lesions to be included in the study will be excluded and immediately treated with the government standard. If the neem and coconut oil mix proves to be more efficacious, these children will be treated with the neem and coconut oil mix as soon as possible (quick analyses will be conducted to enable this to be within one month) should their infections not have cleared. If several eligible lesions are present on one foot, only those (at most two) that allow a clear discernment of the developmental stage of the embedded parasite and a quantification of the inflammatory response around the lesion will be selected for data analysis. Lesions occurring in a cluster and lesions which the patient has attempted to manipulate will not be selected for data analysis.

Patients with the presence of gross inflammation, abscess or ascending lymphangitis or lymphedema on either foot will also be excluded. Children with such complications of tungiasis will be referred to the nearest health facility for treatment.

## Withdrawal criteria

Since the trial is so short, each child only receiving 2 applications of the trial product, two days apart, it is unlikely that any study subject will need to withdraw. However, should a study subject experience adverse effects after the first application they will be expected to report this to their teacher who will contact the Study Investigator. The study investigator and physician will examine the subject and decide whether the effects are due to the study product and if necessary will give appropriate treatment. In this instance, the study subject will be withdrawn from the trial. The withdrawal and reasons will be noted in the Case Report Form. The subject will not receive any further application of either study or control products, but the subject will be monitored for one week and progress recorded in the CRF. A study subject will be withdrawn if they remove the embedded flea or apply any other product during the observation week. The withdrawn subjects will be replaced with another subject if there is another suitable candidate, fitting all inclusion criteria, in the school. If such a subject is identified the caregiver will be contacted and the Informed Consent process and enrolment will proceed as for all other subjects.

# 6. Treatment of study participants

Both feet will be washed and dried thoroughly before examination and applying the interventions. Photographs will be taken of both feet and maps drawn of the location of the individual lesions for observation. These will be kept in the Case Report Forms and are included in Annex 2. After the observations are complete the lesions will be treatedeither with the trial product or with Potassium permanganate and Vaseline.

**The neem and coconut oil mix**: Drops of the neem and coconut oil mix oil will be applied directly to all the embedded fleas on the foot. The neem and coconut oil mix is supplied in a dropper bottle allowing for controlled application. Each case will receive one application on day 1 and on day 3. The oil will be applied, one drop directly onto each of the embedded fleas and spread over the surrounding inflamed skin.

**<u>Potassium permanganate</u>:** This will be applied according to the National Guidelines. The foot will be soaked in 0.05% potassium permanganate in a basin for 15 minutes and then air dried, before applying Vaseline all over the foot.

All study subjects will receive a tetanus vaccination, as per government guidelines.

### Follow up

Each study subject will be assessed on days 3, 5 and 7 after the first treatment. This assessment will include examination by the Trial Physician for inflammation, secondary infection and signs of adverse effects of the Trial Product. In addition, each of the individual embedded fleas selected for the study will be observed for 15 minutes for viability signs.

During the course of the study the subjects will be free to take any medication for other diseases, but not anything that they would normally use to treat themselves for jiggers. During the enrolment process this will be emphasized.

## The Investigational Product

The neem and coconut oil mix comprises the oil extracted from the seeds of two plants: the neem tree (*Azidarachta indica)* and the coconut palm (*Coco nucifera*). The test product will be prepared by a qualified chemist at icipe, at a ratio of 20:80 (neem oil : coconut oil), under sterile conditions.

### Packaging of the Test Product

The neem and coconut oil mix will be packaged in dark dropper bottles under sterile conditions, to minimize the entry of contaminants and to control flow during use, with a label including date of manufacture and expiry.

### Shelf life

There is no stability data available for the investigational product, a mix of pure neem oil and coconut oil. Neem oil must be stored in a cool, dark place and sealed from the air to prevent deterioration. Stored in this way the manufacturer has evaluated the shelf life to be at

least 2 years. Coconut oil has a shelf life of 2 years. For the current study the amount of product needed for the study is very small, a maximum of 100 ml assuming each of the 50 subjects needs 1ml for each of two applications. The amount of product needed will all be from the same batch, prepared within one month of the start of the study. The study will take place over 14 days and will be conducted in a single site, so the active ingredients are unlikely to degrade from the time of preparing the mix, shortly before the trial. Immediately before the mix is prepared we will subject the neem oil to GC-MS to assess the Azadirachtin content and compare it to that submitted here. Once prepared, the 20% mix will also be assayed by GC-MS to obtain Azadirachtin concentration of the actual investigational product.

### Participant's compliance

All applications of the test and control products will be conducted by the study personnel, and does not rely on the subjects' compliance, and thus no compliance monitoring will be necessary. Should a subject be absent from school on the day they are to receive their second dose, or an assessment, the study personnel will make every effort to find the subject through the teachers and community health volunteers associated with the study. They will be asked to visit school for the purpose of the study.

## 7. Assessment of Efficacy

### Primary Outcome:

The embedded fleas will be assessed at baseline and every 2$^{nd}$ day for 7 days for the four viability signs (expulsion of eggs, excretion of faecal thread, excretion of faecal liquid, pulsations/contractions) using the digital handheld video microscope. The observation of each of these signs will be recorded in the case Report Form (Annex 2).

The **primary efficacy parameter** will be the proportion of viable embedded sand fleas which lose viability signs after seven days of follow-up. An embedded sand flea will be considered to be non-viable when none of the four viability signs are detected during 15 minutes of observation on two consecutive follow-up examinations.

Each lesion will be photographed using a macro lens and flash at each assessment.

In addition a **secondary efficacy parameter** will be abnormal development of the embedded sand flea; no increase in size, discolouring or desiccation of the abdominal rear cone.

The observation units will be single sand flea lesions.

### Secondary Outcome:

The study subjects will be examined for the number of embedded fleas, manipulated lesions (scars of extracted fleas) and signs of inflammation around them (not only those for observation). Records will include the total number of embedded viable fleas, manipulated lesions and presence or absence of inflammation in the Case Report Form. Each study subject will also be asked to rate their experience of pain and itching using a Visual Analogue Scale (included in Annex 3).

These other **secondary efficacy parameters** will be:

- the proportion of study subjects with reduced inflammation and
- the proportion of study subjects who report a reduction in their experience of pain and itching on each treated foot.

## 8. Assessment of Safety

Each study subject will be assessed every two days for 7 days after the initial treatment. During this time the Trial Physician will conduct a general physical exam and will examine each subject carefully for localized reactions to the trial and control products, particularly dermatitis. No body fluids or samples will be taken from the study subjects for this trial.

### Adverse Events/Serious Adverse Events

The study will include the following evaluations of safety as described below and according to the time points provided in the Time and Events Schedule. Any clinically significant abnormalities occurring from signing of the ICF onwards until 7 days after the application of the study product must be recorded on the Adverse Event section of the CRF. Thereafter,

reporting will be limited to all serious adverse events. Any clinically significant abnormalities (including those persisting at the end of the study/early withdrawal) will be followed by the investigator until resolution or until a clinically stable endpoint is reached.

The investigators, together with the sponsor's medical monitor, will be responsible for the safety monitoring of the study, and will halt application of the product on further subjects in case of a Serious Adverse Event.

Adverse Events

All adverse events, whether serious or non-serious, will be collected at all visits from signing of the ICF onwards until 14 days after the study product/Neem application.

Thereafter, reporting will be limited to all serious adverse events last study-related procedure. Solicited local and systemic adverse events will be reported by the subject until 7 days after each administration of study product/neem.

Solicited Adverse Events

Solicited adverse events are precisely defined events that subjects are specifically asked about and which are noted by subjects in the diary. The subjects will be closely observed by study-site personnel for the first 30 (±10) minutes after each administration of study product, and any unsolicited, solicited local or systemic adverse events will be documented during this period. Upon discharge from the site, subjects will receive a diary to record solicited local reactions. Subjects will be instructed to record solicited local and systemic adverse events in the diary in the evening after each study product administration and then daily for the next 7 days. The diary needs to be completed on site at next subject visit and before the subject leaves the site. The investigator should discuss the information from the diary with the subject, document the relevant information in the clinic chart, and complete the relevant parts of the CRF.

On-site and diary reported solicited adverse events will be captured on a separate CRF page as described in the CRF Completion Guidelines, in contrast to the unsolicited adverse events

which will be reported on the Adverse Event page of the CRF. The investigator must record in the CRF his/her opinion concerning the relationship of the adverse event to the study product.

Solicited Local topical product application area Adverse Events

Subjects will also be instructed on how to note occurrences of erythema, induration/swelling, pain/tenderness and itching at the application site in the evening after each administration of the study product and then daily for the next 7 days in the diary at approximately the same time each day.

Subjects will also be instructed on how to note the following symptoms in the evening after each administration and then daily for the next 7 days in the diary at approximately the same time each day:

- Nausea/vomiting
- Arthralgia
- Fever
- Headache
- Fatigue/malaise
- Myalgia
- Chills

**Adverse events treatment**

If any subject should have a reaction to the study product the Trial Physician will detect it and will take the subject to the nearest health centre for appropriate care.

**Adverse events facilities**

The nearest health facility that will be used for referral of adverse events is Gede Health Centre, a 10 to 15 minute drive from where the study will take place.

# 9. Statistics

## 9.1 DSMB

This trial will not have a DSMB since it will take place at a single site, is of short duration and involves only a few investigators.

## 9.2 Sample Size

A superiority trial will be implemented hypothesizing that the neem and coconut oil mix treatment is better than the standard government recommended treatment. The percentage of fleas killed by either treatment, or the percentage of patients that meet the primary outcome definition (e.g. percentage therapeutic success) is compared between two randomised groups.

The sample size was estimated using a calculator designed for binary outcomes in parallel group superiority trials (https://www.sealedenvelope.com/power/binary-superiority/). Accordingly, 40 patients (with at least one viable embedded flea) are required in each group (total 80 patients) to have a 80% chance of detecting, as significant at the 5% level, an increase in the primary outcome measure from 38% in the control group (as previously published for potassium permanganate) to 68% in the experimental group (as estimated by community reports). One hundred patients will be recruited to allow for loss at follow up.

## 9.3. Sampling procedures

Schools will be selected from all the schools in Kilifi County that haves not been exposed to recent tungiasis control efforts, is known to have a high prevalence of tungiasis and teachers and caregivers are willing to participate. A school caregivers and teachers meeting for the appropriate year groups will be held to explain the study prior to any survey. A survey of all children's feet in the appropriate age group, both boys and girls, will be done to identify those with appropriate infections for inclusion in the study. Once potential study subjects have been identified according to the inclusion/exclusion criteria, the caregivers will be informed and invited for a meeting. The study will be explained to the caregivers and the children again and both will be requested to give informed consent and assent. If children refuse to give assent, even if caregivers give their consent, the children will not be forced to participate. More schools may need to be recruited into the survey depending on the availability of willing children with appropriate lesions for inclusion. However, the study staff will be the same in all sites. Children identified with jiggers, but who do not meet the inclusion criteria

will be offered immediate treatment with the government standard (potassium permanganate).

## 9.4. Randomization Process

The assignment of subjects to a treatment will be done by drawing a card from an envelope for the first case. Each subsequent case will revceive alternate products.

## 9.5. Data Management

Data regarding the case, the flea viability, inflammation, pain and itching will be documented on Case Report Forms which will be pre-tested in pilot sites before the study commences. These tools have been adapted from past studies conducted by Professor Feldmeier of Universite Charite, Berlin, Germany (14) with Zanzarin and Dimeticone NYDA® and are included in the Annexes. Completed CRFs will be stored in a locked room away from the study site.

Each evening data collected will be verified and entered into a data base. Double data entry will be used to check for errors during entry. Any spurious data will be checked with the original Case Report Forms and corrected if possible.

For statistical analysis, data of individuals will be anonymized, using patient code numbers only, no names. The databases will be backed up each night and will be remote from the study site. Only the Investigators will have access to the CRFs and databases.

Records for the active ERC approved research project will be stored in a secure location (locked filing cabinets in locked room). Study information containing personal identifiers stored on computers and other storage devices will be password protected to prevent unintentional breaches of confidentiality in the event the storage device is lost or stolen. Similarly, paper records identifying research subjects including consent forms will be kept under the personal control of the core researchers. Consent forms and paper data records will be locked in file cabinets in offices at the performance site and at icipe-Nairobi. Electronic Data Records will be stored in password protected files, on icipe maintained

servers with regular back-up. Research data will be retained for 3 years after the completion of the project. Records may be preserved in hard-copy, electronic or other media form, and will be accessible for audit purposes. Records for completed projects will be stored in secure locations at icipe with the same care used when the project was active.

### 9.6. Data Analysis

Data will be analysed using generalized linear mixed models, including random (patient ID) and fixed effects (treatment, potential confounders like age group, sex, overall status of infection). Proportion data will be modelled with the binomial distribution fitted. Any missing values will be excluded from the analyses.

*We will be comparing the following parameters for the trial and control groups (n=50 in each group):*

1. *Proportion of fleas killed*
2. *Proportion of fleas with abnormal development*
3. *Proportion of fleas with loss of inflammation*
4. *Proportion of subjects with reduced pain score*
5. *Proportion of subjects with reduced itching score*

There will be no interim analyses of the data since the trial period is only a few weeks. Any deviation from the original statistical plan will be described and justified in the final report.

Should any violations of the protocol be identified by the Investigators or Monitor, these will be reported to the PPB ECCT and KEMRI-SERU within 1 month of the end of the study.

## 10. Direct Access to Source Data/Documents

The Investigators will permit inspections from PPB including direct access to source data/documents.

# 11. Quality Control and Quality Assurance

## 11.1. The Investigators

Each of the Investigators is qualified and experienced to conduct their role in the trial as described here and their full CVs are provided in the Annexes.

**Dr. Ulrike Filinger (co-PI)**: Project oversight at icipe. Study design and preparation of protocol, investigation of secondary outcomes of treatment on fleas (e.g. reduction of egg-laying), statistical data analyses and review of manuscript.

Ulrike Fillinger is a senior scientist/ public health entomologist at icipe and a lecturer at the London School of Hygiene and Tropical Medicine. She has been PI and co-PI on multiple research grants in the field of public health entomology with a focus on mosquito and malaria control. She has supervised numerous PhD and Masters students and published international peer-reviewed journals. Ulrike has been responsible for the design, monitoring and evaluation of mosquito control program in three countries. She has lived and worked with icipe in Kenya since 2000. Ulrike has an MSc and a PhD from the University of Heidelberg, Germany.

**Dr. Lynne Elson (co-PI)**: Planning of project, study design and preparation of protocol, training & coordinating CHW in collaboration with the clinical officer, Dr. Askaa Moraa (below), for treatment application, parasitological examinations; management of documentation forms and data, local supervision of data entry, writing of manuscripts.

Lynne Elson is an independent Public Health consultant, with her own work permit and has been working in Kenya since 1995. She has conducted research and has published in peer-reviewed journals on leishmaniasis, lymphatic filariasis, onchocerciasis, schistosomiasis, HIV, gender-based violence and child vulnerability. She currently consults for International NGOs such as PATH (Program of Appropriate Technology in Health) and EGPAF (Elizabeth Glaser Pediatric AIDS Foundation) focussing on program planning, monitoring and evaluation, proposal and report writing covering HIV/AIDS, TB, malaria, RH/FP and MNCH. In 2012, on a voluntary basis, Lynne supported a grass-roots project with the Community Health Workers in Malindi sub-county, to assist them to relieve the suffering inflicted by tungiasis in their community.

Lynne was part of a team who developed the "Kenya Policy Guidelines on Jigger Prevention and Control, 2014".

Lynne has a BSc from Imperial College London, a PhD from Liverpool School of Tropical Medicine, UK and an MPH from James Cook University, Australia.

**Dr. Patrick Sawa:** clinical examinations, treatment and monitoring of adverse events, reporting of adverse events, referral of complicated tungiasis and other cases not eligible for enrolment, review of protocol and manuscript.

Dr Sawa has been the Medical Officer in charge of a rural outpatient clinic since 2002, providing health services to the local community in Mbita and managing clinical research projects for icipe. In this time he has been the Study Physician for 9 clinical trials from which 12 MSc and 5 PhD students have earned their degrees. He has acted as Malaria Programme Coordinator for ICIPE-Mbita for 18 months and deputized the Station Manager for 4 years. Patrick gained an MBChB from Moi University, Eldoret in 1998, an M.Sc. in Infectious Diseases from the University of London in 2008 and a Diploma in Tropical Medicine from the London School of Hygiene and Tropical Medicine in 2009.

**Askaa Moraa**: clinical examinations, treatment and monitoring of adverse events, referral of complicated tungiasis and other cases not eligible for enrolment, review of manuscript.

Askah Moraa is a Clinical Officer with 10 years of clinical experience working for the Kenyan Ministry of Health, in health centres and hospitals around the country. She is currently the Clinical Officer in charge of the Comprehensive Care Centre at Gede Health Centre and is also studying for a Diploma in Public Health at Pwani University.

**Mr. Sammy Baya**: coordination of field work, mobilising caregivers and subjects, obtaining informed consent/assent, treatment of subjects and all cases at end of study. He will be the main contact person for the caregivers and subjects.

Sammy Baya has been the lead Community Health Worker for Dabaso Community Unit since 2005. He has coordinated the 50 CHWs, collected and collated their reports and submitted them to MOH on a monthly basis. He has been the key driving force behind registration as a legal organization, expanding activities and initiating the jigger project. He is greatly respected within the community.

## 11.2. Monitors and monitoring plan

The trial will contract an Independent Monitor from ClinWin Research Services, Nairobi for Quality Assurance, to ensure compliance to the protocol and ultimately the validity of the data.

ClinWin are a mid-sized Contract Research Organization (CRO) based in Nairobi, providing outsourced clinical development and consulting services to biopharmaceutical companies, academic medical centres, investigator sites, not-for profit research organizations and Government. The have over 10 years' experience in clinical trials management. The Director,

Nicodemus will himself be the study monitor for this trial, is a member of the Association of Clinical Research Professionals of USA and The Institute of Clinical Research of UK.

The Independent Monitor will review the protocol, SOPs and all study documents, participate in the training and ensure the study is set up correctly, ensure consent procedures are properly conducted, SOPs adhered to and data recorded and then entered into the database correctly. The Monitor will conduct site close-out.

Should the ECCT or KEMRI/SERU wish to visit they will be welcome to do so and will have access to all documents and electronic files.

## 12. Ethics

The principles set out in the WHO International Ethical Guidelines for Epidemiological Studies[2] will be followed. The study team will treat all subjects with respect. Participation will be voluntary and the team will ensure subjects, teachers and caregivers are aware of this. Subjects may withdraw from the study at any time.

The project will ensure that consents for participation are only given after the caregivers and children have been comprehensively informed of the purpose of the study and they understand the activities in which they will participate and their implications. The explanations will be given in the local language (Giriama) by a trained member of the project. A document written in English and Giriama will be handed in to the participant to read and digest before being given the Informed Consent and Assent Documents (included in the Annexes) for signing in agreement to participate. An impartial witness will be assigned to attest that the information in the consent and assent forms and any other written information were accurately explained to and apparently understood by the participant and that consent/assent was freely given.

### Recruitment Strategy

One school will be selected from all the schools in Kilifi County that were not exposed to recent tungiasis control efforts, are known to have a high prevalence of tungiasis and teachers and caregivers are willing to participate. A school caregivers and teachers meeting for the appropriate year groups will be held to explain the study prior to any survey. A survey of all children’s feet in the appropriate age group, both boys and girls will identify those with

[2] http://www.ufrgs.br/bioetica/cioms2008.pdf

appropriate infections for inclusion in the study according to the inclusion and exclusion criteria. Although only 100 children will be needed, a list of all potential study subjects will be created. If more than 100 children meet the inclusion criteria, 100 will be selected randomly. All excluded, will be treated with the standard government treatment.

### Subject Approach

Once potential study subjects have been identified, the caregivers will be informed and called for a meeting. The study will be explained to the caregivers and the children again using appropriate language. In private the caregivers will be given to read, or will be read the consent form in the local language (Giriama) and asked to sign the consent forms. The children (subjects) will be read the assent form which uses simple language and will be asked to 'sign' the assent form. If children refuse to give assent, even if caregivers give their consent, the children will not be forced to participate. More schools may need to be recruited into the survey depending on the availability of willing children with appropriate lesions for inclusion.

### Non-coercive contact

Potential subjects and their caregivers will be informed orally and in writing that they are free to reject participation, as well as to withdraw from the study at any moment; they will be informed that by doing so they will be exercising their rights and will not suffer any detriment.

If the participant and caregiver are unable to read and/or write, an impartial witness will be present during the consent discussion. After the written informed consent form is read and explained to the participant and caregiver, and after he or she has orally consented to participate in this study, and has either signed the consent form or provided his or her fingerprint, the witness must sign and personally date the consent form. By signing the consent form, the witness attests that the information in the consent form and any other written information was accurately explained to, and apparently understood by, the participant, and that consent was freely given.

Children eligible to participate in the study will be informed in simple language in presence of the candidate's caregiver, and with the help of an interpreter. Children able to read will be requested to assent the procedure by making a mark on the assent form. Children for who informed consent has been obtained from the caregivers or guardians, but manifestly refuse to participate will be excluded from the study.

## 12.2. Treatment and/or management after completion of trial

The initial parents' meeting to explain the study will include information on prevention of new infections so that families will be able to protect their children after the study. At the end of the study, once analyses are complete, all children in the school will be treated with the product which is found to be the most effective. A parents' meeting will be called to provide them with results of the study and to re-inforce methods of prevention and appropriate treatment.

## 12.3. Confidentiality

The study field team will only include the Investigator, the Physician, the Community Coordinator and 2 Community Health Volunteers (CHVs). All but the CHVs have completed a Human Subjects in Research course and have been involved in the development of the protocol. It will be the responsibility of the Investigator to ensure all team members understand the protocol and the issue of patient confidentiality and abide by the KEMRI, PPB and WHO International Ethical Guidelines. The Community Coordinator will brief the CHVs who will only be involved in tracing and washing subjects' feet, and will never have access to any of the Case Report Forms.

## 12.4. Follow-up of trial study participants

Other than the 7 days of follow up and assessments of the study subjects, there will be no further follow up.

## 12.5. Insurance and indemnity measures

The investigators and participants will be covered by an insurance policy held by the sponsor (icipe) which will be set up immediately after approval of the protocol and before implementing the study. Icipe is already in the process of discussing this trial with the insurers. The investigators will submit the insurance certificates to PPB on receipt from the insurance company.

## 13. Data Handling and Record Keeping

Data regarding the case, the flea viability, inflammation, pain and itching will be documented on Case Report Forms which will be pre-tested in pilot sites before the study commences. These tools have been adapted from past studies conducted by Professor Feldmeier of Universite Charite, Berlin, Germany (14) with Zanzarin and Dimeticone NYDA® and are included in the Annexes. Completed CRFs will be stored in a locked room away from the study site.

Each evening data collected will be verified and entered into a data base. Double data entry will be used to check for errors during entry. For statistical analysis, data of individuals will be anonymized, using patient code numbers only, no names. The databases will be backed up each night and will be remote from the study site. Only the Investigators will have access to the CRFs and databases.

### Data Storage

Records for the active ERC approved research project will be stored in a secure location (locked filing cabinets in locked room). Study information containing personal identifiers stored on computers and other storage devices will be password protected to prevent unintentional breaches of confidentiality in the event the storage device is lost or stolen. Similarly, paper records identifying research subjects including consent forms will be kept under the personal control of the core researchers. Consent forms and paper data records will be locked in file cabinets in offices at the performance site and at icipe-Nairobi.

Electronic Data Records will be stored in password protected files, on icipe maintained servers with regular back-up. Research data will be retained for 3 years after the completion of the project. Records may be preserved in hard-copy, electronic or other media form, and will be accessible for audit purposes. Records for completed projects will be stored in secure locations at icipe with the same care used when the project was active.

## 14. Publication Policy

The results of the study will be published in a high-ranked peer-reviewed international journal. The results will be also shared with the participating communities through school meetings, and with policy makers and stakeholders in the County through a stakeholder meeting. The results will also be presented in national and international conferences for Tropical Diseases.

# 15. Bibliography


1. Nagy N, Abari E, D'Haese D, Calheiros C, Heukelbach J, Mencke N, et al. Investigations on the life cycle and morphology of Tunga penetrans in Brazil. Parasitol Res. 2007;: p. 101 (Suppl 2):S233–S242.

2. Heukelbach J, Costa A, Wilcke T, Mencke N, Feldmeier H. The animal reservoir of Tunga penetrans in severely affected communities of north-east Brazil. Med Vet Entomol. 2004;: p. 18: 329–335.

3. Cooper J. Tunga penetrans infestation in pigs. Veterinary Records. 1976;: p. 98:472.

4. Cooper J. An outbreak of Tunga penetrans in a pig herd. Veterinary Records. 1967;: p. 80: 365–366.

5. Heukelbach J, de Oliveira F, Hesse G, Feldmeier H. Tungiasis: a neglected health problem of poor communities. Trop Med Int Health. 2001;: p. 6: 267–272.

6. Bezerra S. Tungiasis—an unusual case of severe infestation. Int J Dermatol. 1994;: p. 33:725.

7. Cardoso A. Generalized tungiasis treated with thiabendazole. Arch Dermatol. 1981;: p. 117:127.

8. Feldmeier H, Heukelbach J, Eisele M, Sousa A, Barbosa L, Carvalho C. Bacterial superinfection in human tungiasis. Trop Med Int Health. 2002;: p. 7: 559–564.

9. Joseph J, Bazile J, Mutter J, Shin S, Ruddle A, Ivers L, et al. Tungiasis in rural Haiti: a community-based response. Trans R Soc Trop Med Hyg. 2006;: p. 100: 970–974.

10. Tonge B. Tetanus from chigger flea sores. J Trop Pediatr. 1989;: p. 35: 94.

11. Feldmeier H, Eisele M, Saboia-Moura R, Heukelbach J. Severe tungiasis in underprivileged communities: case series from Brazil. Emerg Infect Dis. 2003;: p. 9: 949–955.

12. Feldmeier H, Sentongo E, Krantz I. Tungiasis (sand flea disease): a parasitic disease with intriguing challenges for public health. Eur J Clin Microbiol Infect Dis. 2012;: p. 32:19–26.

13. Heukelbach J, Bonow I, Witt L, Feldmeier H, Fischer P. High infection rate of Wolbachia endobacteria in the sand flea Tunga penetrans from Brazil. Acta Trop. 2004; 92(3): p. 225-30.

14. Thielecke M, Nordin P, Ngomi N, Feldmeier H. Treatment of Tungiasis with Dimeticone: A Proof-of-Principle Study in Rural Kenya. PLOS Negl Trop Dis. 2014;: p. 8(7): e3058. doi:10.1371/journal.pntd.0003058.

15. Heukelbach J, Pilger D, Oliveira F, Khakban A, Feldmeier H. A highly efficacious pediculicide based on dimeticone: Randomized. BMC Infectious Diseases. 2008;: p. 8:115.

16. Feldmeier H, Kehr J, Heukelbach J. A plant-based repellent protects against Tunga penetrans infestation and sand flea disease. Acta Trop. 2006 Oct; 99(2-3): p. 126-36.

17. Gitau AK, Oyieke FA, Evan M. Efficacy of Coconut Oil in The Control of Acute Tungiasis. International Journal of New Technology and Research. 2015 Sept; 1(5): p. 85-92.

18. BOSTID. Neem: A Tree for Solving Global Problems Council NR, editor.: National Academies Press; 1992.

19. Abdel-Ghaffar F, Semmler M. Efficacy of neem seed extract shampoo on head lice of naturally infected humans in Egypt. Parasitol Res. 2007;: p. 100:329–332.

20. Abdel-Ghaffar F, Al-Quraishy S, Sobhy H, Semmler M. Neem seed extract shampoo, Wash Away Louse, an effective plant agent against Sarcoptes scabiei mites infesting dogs in Egypt. Parasitol Res. 2008;: p. 104(1):145-8.

21. Deng Y, D S, Yin Z, Guo J, Jia R, Xu J, et al. Acaricidal activity of petroleum ether extract of neem (Azadirachta indica) oil and its four fractions separated by column chromatography against Sarcoptes scabiei var. cuniculi larvae in vitro. Exp Parasitol. 2012. 2012;: p. 130(4):475-7.

22. Tabassam S, Iqbal Z, Jabbar A, Sindhu Z, Chattha A. Efficacy of crude neem seed kernel extracts against natural infestation of Sarcoptes scabiei var. ovis. J Ethnopharmacol. 2008;: p. 17;115(2):284-7.

23. Srivastava R, Ghosh S, Mandal D, Azhahianambi P, Singhal P, Pandey N, et al. Efficacy of Azadirachta indica extracts against Boophilus microplus. Parasitol Res. 2008. 2008;: p. 104(1):149-53.

24. Guerrini V, Kriticos C. Effects of azadirachtin on Ctenocephalides felis in the dog and the cat. Vet Parasitol. 1998; 74(2-4): p. 289-297.

25. Okumu F, Knols B, Fillinger U. Larvicidal effects of a neem (Azadirachta indica) oil

formulation on the malaria vector Anopheles gambiae. Malaria Journal. 2007;: p. 6:63.

26. Mulla M, Su T. Activity and biological effects of neem products against arthropods of medical and veterinary importance. J Am Mosq Control Assoc. 1999. 1999;: p. 15(2):133-52.

27. Gianotti R, Bomblies A, Dafalla M, Issa-Arzika I, Duchemin JB, Eltahir EA. Efficacy of local neem extracts for sustainable malaria vector control in an African village. Malaria Journal. 2008;: p. 7:138.

28. Biswas K, Chattopadhyay I, Banerjee R, Bandyopadhyay U. Biological activities and medicinal properties of neem (Azadirachta indica). Current Science. 2002; 82(11: 1336-1345).

29. H S. Properties and Potential of Natural Pesticides from the Neem Tree, Azadirachta Indica. Annual Review of Entomology. 1990; 35: p. 271-297.

30. Boeke SJ, Boersma MG, Alink GM, van Loon JJA, van Huis A, Dicke M, et al. Safety evaluation of neem (Azadirachta indica) derived pesticides. J Ethnopharm. 2004; 94: p. 25–41.

31. Ruskin FR. Neem: A tree for solving global problems. Report of an Ad Hoc Panel of the Board of Science and Technology for International Development, National Research Council. In Sciences NAo, editor..: National Academic Press; 1992.

32. Deng Y, Cao M, Shi D, Yin Z, Jia R, Xu J, et al. Toxicological evaluation of neem (Azadirachta indica) oil: acute and subacute toxicity. Environ Toxicol Pharmacol. 2013 Mar. 2013 Mar; 35(2): p. 240-6.

33. Wang C, Cao M, Shi D, Yin Z, Jia R, Wang K, et al. A 90-day subchronic toxicity study of neem oil, a Azadirachta indica oil, in mice. Hum Exp Toxicol. 2013 Sep. 2013 Sep; 32(9): p. 904-13.

34. Lai S, Lim K, Cheng H. Margosa oil poisoning as a cause of toxic encephalopathy. Singapore Med J. 1990 Oct; 31(5): p. 463-5..

35. Sinniah D, Baskaran G. Margosa oil as a cause of Reye's syndrome. Lancet. 1981; 1: p. 487-9.

36. Bond C, Buhl K, Stone D. Neem Oil General Fact Sheet. National Pesticide Information Center, Oregon State University Extension Services.; 2012.

37. Agency UEP. EPA. [Online].; 2010 [cited 2016 August. Available from: https://www3.epa.gov/pesticides/chem_search/reg_actions/registration/fs_PC-025006_07-Apr-10.pdf.

38. Beltrán E, Ibáñez M, Gracia-Lor E, Sancho J, Hernández F, Thompson DG. Application of liquid chromatography/mass spectrometry in assessment of potential use of azadirachtins (TreeAzin TM ) against Asian longhorned beetle. Anal. Methods. 2014; 6(19): p. 8063–8071.
