# Supplementary material for: Efficacy of a mixture of neem seed oil (Azadirachta indica) and coconut oil (Cocos nucifera) for topical treatment of tungiasis. A randomized controlled, proof-of-principle study
Source: PLoS Negl Trop Dis. 2019 Nov 22;13(11):e0007822. doi: 10.1371/journal.pntd.0007822 (PMC6897421; doi:10.1371/journal.pntd.0007822)
Supplement: S2 Annex — (PDF) [file pntd.0007822.s002.pdf]

Annex 2B: Patient Information Leaflet and Informed Consent Form-English

Study title: "Randomised Controlled Trial of a locally-made, herbal remedy for treatment of jiggers (*Tunga penetrans)*"

Name of the coordinating institution: Dabaso Tujengane Community Based Organization

Primary Objective: To determine whether the neem and coconut oil mix kills a higher proportion of embedded jiggers than the current standard of potassium permanganate and Vaseline, in 7 days.

Secondary Objectives:

1. To determine whether the neem and coconut oil mix reduces inflammation, pain and itching better than the standard treatment, in 7 days.
2. To determine the safety of the neem and coconut oil mix for topical use.

Background

Your child has been identified as having jiggers in his/her feet. Jiggers are a flea which burrows into the skin and grows rapidly causing your child pain and itching. We are trying to find a simple, safe, pain-free and affordable way to treat jiggers. The neem and coconut oil mix is a local remedy. It seems that it is good at killing jiggers but we must prove it. The government recommends a chemical called Potassium permanganate which we will also use. As far as we know, both products are safe and cause no harm. We will compare the two to find out if the neem and coconut oil mix is better than Potassium permanganate at killing jiggers in the feet of children.

The study

Children age 6-14 years will be recruited into the study if they have at least one live jigger which is clear for the researchers to observe. The children will have to leave their class for about 30 minutes every 2 days for 1 week for the investigators to observe them.

Your child will be treated either with permanganate OR with the neem and coconut oil mix. The selection of medicine to be used for your child is random. The doctor will make careful observations about the jiggers on the first day and every other day for 7 days. The doctor will also ask your child about how much pain and itching they are feeling. At the end of the 7 days we will treat your child with the treatment that works best if any jiggers have survived on any foot.

It is **VERY** important that you and your child, **DO NOT** cut out any jiggers from the child's foot during this time, nor put any other medicines or chemicals on them.

The study will not be taking any samples from the child. We will only make observations of the jiggers. The information we collect from the child will be entered into a computer

together with that from all of the children that we treat. Someone with special training will analyse the information. The forms will be kept in a locked office away from your home and school. It is important that we have your names so that the researchers can come back to observe the child each visit.

While we will not share individual information, we will come back to tell you about the results of the tests. We will also share these findings with other organisations and to the Kilifi County and the National Ministry of Health.

If your child is found to have any other disease we will advise you on the best way to manage it. No one will be given anything for participating in the study, except treatment of their jiggers.

The study has been approved by the KEMRI Ethical Review Committee.

Risks

The participation in the study does not present any health risk to the participant. There is no specific risk concerning the application of the neem and coconut oil mix or potassium permanganate to the skin. The products are not toxic.

Another possible risk is that your child may be teased for having jiggers since this will be revealed to other children in the school by participating in the study. The Researchers will do all they can to avoid this.

Right of refusal

The decision to participate in the study is voluntary. Clinical examination and treatment will be conducted in the school. The Kilifi County Department of Health, the Department of Education and the school principal have been informed about the study and support it.

You are absolutely free to accept or to refuse the participation of your child in the study without any consequences. If you agree for your child to participate, but your child refuses, we will not force them to do so, and you must not force them either. ALL children with jiggers in the school will be treated after the study is complete with the product found to be the best, whether they participated or not.

Contacts

For further information concerning your child’s participation, please contact the following person:

Mr. Sammy Baya, Chairman of Dabaso Tujengane CBO on telephone number: 0729 678338

or

Prof. Elizabeth Bukusi, KEMRI Scientific and Ethical Review Unit, KEMRI, Nairobi : 020 2722541, 020 2713349, 0722-205901, 0733-400003

Approval

The undersigned … (name and first name) testifies that she/he is the legal guardian of the child ……………………………………
…...……………………………(name of child) and that she/he has read and understood the consent form which was also read aloud and explained by .. …

I understand the objectives, the necessities, the potential risks and advantages regarding the participation of my child in the study, including that my child will have to leave his/her class for 30 minutes every 2 days for one week and will have to return at the weekend.

I agree that living sand fleas remaining at the end of the study are treated with the method shown to be the most effective.

I was given the guarantee that all information which could lead to an identification of my child will be kept strictly confidential and will be maintained in a database saved by a password. I agree that all doctors or scientists participating in the study have access to this information. Upon request, I will have access to my child's data which - on my demand - will be destroyed by the investigators. I voluntarily accept the participation in this study based on these conditions. I have the right to withdraw my child from the study at any time without giving any justification for the removal. I only have to give a short verbal or written statement. The removal will not have any negative influence on actual or future treatments provided by the local clinics or Dabaso Tujengane CBO.

School … … … … … … … … … … … … … … ………………………… Date: .... … … …

Subject Study ID-no…………………………………

Parent/Caregiver

Name:... … … … … … … … … .... Signature……………………………………

Date…………………………………

Investigator who provided the information:

Name:... … … … … … … … … .... Signature……………………………………

Date…………………………………

Witness:

Name:... … … … … … … … … .... Signature……………………………………

Date…………………………………

Annex 2C: Patient Information Leaflet and Informed Assent Form for Study Subjects-English

Study title: "Randomised Controlled Trial of a locally-made, herbal remedy for treatment of jiggers (*Tunga penetrans)*"

Name of the coordinating institution: Dabaso Tujengane Community Based Organization

We are trying to show that this medicine (*show bottle)* the neem and coconut oil mix can kill the jiggers in your feet in 7 days without causing you any pain or other problems, and that it is better at doing this than the purple medicine called potassium permanganate, which some people use. If you decide that you want to be part of this study, you will be asked to sign this form (write your name on this form) and allow us to:

a. wash your feet,
b. examine them carefully,
c. ask you questions about how the jiggers make you feel,
d. watch your jiggers through a special camera,
e. take photographs of your feet
f. put the medicine, which does not hurt, on your jiggers. We will either put the neem and coconut oil mix drops on the jiggers in your foot, or you will put your feet into a basin of the purple medicine for 15 minutes.

We expect this will take around half an hour. Then we will ask you to let us examine your jiggers again every 2 days for 7 days, that's 4 times. This will include once on the weekend.

There are some things about this study you should know. The medicine will not hurt you but the purple medicine will change the colour of your foot. This colour will go away after a few days. Other students in school will know that you have jiggers because you came to us and they may make fun of you. We will do everything we can to prevent this.

You will benefit by getting your jiggers treated, but if you do not want to participate then your jiggers will be treated at the end of the study along with all the other students with jiggers.

When we are finished with this study we will write a report about what was learned. This report will not include your name or that you were in the study.

You do not have to be in this study if you do not want to be, even if your parents and teacher said it is okay for you to be in the study. If you decide to stop after we begin, that's okay too. Your parents know about the study too.

If you decide you want to be in this study, please sign your name.

I, _________________________________, want to be in this research study.

___________________________________ ______

(Sign your name here) (Date)

Investigator who provided the information:

Name:... ... ... Signature……………………………………

Date…………………………………

Witness:

Name:... ... ... Signature……………………………………

Date…………………………………
