# Supplementary material for: Efficacy of a mixture of neem seed oil (Azadirachta indica) and coconut oil (Cocos nucifera) for topical treatment of tungiasis. A randomized controlled, proof-of-principle study
Source: PLoS Negl Trop Dis. 2019 Nov 22;13(11):e0007822. doi: 10.1371/journal.pntd.0007822 (PMC6897421; doi:10.1371/journal.pntd.0007822)
Supplement: S3 Annex — (PDF) [file pntd.0007822.s003.pdf]

Case Report Form

Version 4, September 2017

A: Cover Page

School_______________________________

Case Name____

Age________ Sex (m/f) ____ Class _________

Subject Study ID-no.______________

At Baseline: Date__________________

	Left foot	Right foot
Total no. of viable lesions (stage II & III)		
Total no. of lesions to be monitored		

Treatment received____________________________(PP/NC)

CRF-B: Medical History and Physical Exam

Study ID-no.______________

Height____________________ Weight ______________________

Medical History

____
____
____
____
____

Physical Exam

Date	Study Day	Blood pressure	Pulse	Temp	Comments
	1				
	3				
	5				
	7				

Phase IIa, randomised controlled trial of a locally-made, herbal remedy (neem & coconut oil) for treatment of jiggers (*Tunga penetrans*).

Protocol ID: KEMRI-SERU Non-SSC Protocol No. 514, PPB/ECCT/16/05/03/2016(94)

CRF-C1: Pathology

Study ID-no.______________ **Day 1** Date_____/______/ __________

Visual scales:

		Left foot	Right foot
	**Spontaneous pain** (0: not at all, 1: only a little, 2: quite a lot, 3: very much)		
	**Itching** (0: not at all, 1: only a little, 2: quite a lot, 3: very much)		

Examination:

Left foot

		toe 1	toe 2	toe 3	toe 4	toe 5	heel	lateral side	medial side	sole
**acute** pathology	erythema									
	warmness									
	edema									
	desquamation									
	fissure									
	suppuration									
	ulcer									
	abscess									

Right foot :

		toe 1	toe 2	toe 3	toe 4	toe 5	heel	lateral side	medial side	sole
**acute** pathology	erythema									
	warmness									
	edema									
	desquamation									
	fissure									
	suppuration									
	ulcer									
	abscess									

CRF-C3: Pathology

Study ID-no.______________ **Day 3** Date_____/______/ __________

Visual scales:

		**Left foot**	**Right foot**
	**Spontaneous pain** (0: not at all, 1: only a little, 2: quite a lot, 3: very much)		
	**Itching** (0: not at all, 1: only a little, 2: quite a lot, 3: very much)		

Examination:

Left foot

		toe 1	toe 2	toe 3	toe 4	toe 5	heel	lateral side	medial side	sole
**acute** pathology	erythema									
	warmness									
	edema									
	desquamation									
	fissure									
	suppuration									
	ulcer									
	abscess									

Right foot :

		toe 1	toe 2	toe 3	toe 4	toe 5	heel	lateral side	medial side	sole
**acute** pathology	erythema									
	warmness									
	edema									
	desquamation									
	fissure									
	suppuration									
	ulcer									
	abscess									

Phase IIa, randomised controlled trial of a locally-made, herbal remedy (neem & coconut oil) for treatment of jiggers (*Tunga penetrans*).

Protocol ID: KEMRI-SERU Non-SSC Protocol No. 514, PPB/ECCT/16/05/03/2016(94)

CRF-C5: Pathology

Study ID-no.______________ **Day 5** Date_____/______/ __________

Visual scales:

		Left foot	Right foot
	**Spontaneous pain** (0: not at all, 1: only a little, 2: quite a lot, 3: very much)		
	**Itching** (0: not at all, 1: only a little, 2: quite a lot, 3: very much)		

Examination:

Left foot

		toe 1	toe 2	toe 3	toe 4	toe 5	heel	lateral side	medial side	sole
**acute** pathology	erythema									
	warmness									
	edema									
	desquamation									
	fissure									
	suppuration									
	ulcer									
	abscess									

Right foot :

		toe 1	toe 2	toe 3	toe 4	toe 5	heel	lateral side	medial side	sole
**acute** pathology	erythema									
	warmness									
	edema									
	desquamation									
	fissure									
	suppuration									
	ulcer									
	abscess									

CRF-C7: Pathology

Study ID-no.______________ **Day 7** Date_____/______/ __________

Visual scales:

		Left foot	Right foot
	**Spontaneous pain** (0: not at all, 1: only a little, 2: quite a lot, 3: very much)		
	**Itching** (0: not at all, 1: only a little, 2: quite a lot, 3: very much)		

Examination:

Left foot

		toe 1	toe 2	toe 3	toe 4	toe 5	heel	lateral side	medial side	sole
**acute** pathology	erythema									
	warmness									
	edema									
	desquamation									
	fissure									
	suppuration									
	ulcer									
	abscess									

Right foot :

		toe 1	toe 2	toe 3	toe 4	toe 5	heel	lateral side	medial side	sole
**acute** pathology	erythema									
	warmness									
	edema									
	desquamation									
	fissure									
	suppuration									
	ulcer									
	abscess									

CRF-D1: Lesion Viability Scores

Study ID-no.______________ **Day 1** Date_____/_____/ ______Time_______

Right foot: No. of viable lesions______ No. of manipulated lesions_______

	No. 1	No. 2
localisation		
stage		
excretion of faeces (threads)		
excretion of faeces (liquid)		
expulsion of eggs		
pulsation of the flea		
other observations		

Photo Nos. : ____

Left foot: No. of viable lesions______ No. of manipulated lesions_______

	No. 1	No. 2
localisation		
stage		
excretion of faeces (threads)		
excretion of faeces (liquid)		
expulsion of eggs		
pulsation of the flea		
other observations		

Photo Nos. : ____

CRF-D3: Lesion Viability Scores

Study ID-no.____________ **Day 3** Date_____/_____/ ______Time_______

Right foot: No. of viable lesions______ No. of manipulated lesions_______

	No. 1	No. 2
localisation		
stage		
excretion of faeces (threads)		
excretion of faeces (liquid)		
expulsion of eggs		
pulsation of the flea		
other observations		

Photo Nos. : ____

Left foot: No. of viable lesions______ No. of manipulated lesions_______

	No. 1	No. 2
localisation		
stage		
excretion of faeces (threads)		
excretion of faeces (liquid)		
expulsion of eggs		
pulsation of the flea		
other observations		

Photo Nos. : ____

CRF-D5: Lesion Viability Scores

Study ID-no._____________ **Day 5** Date_____/_____/ ______Time_______

Right foot: No. of viable lesions______ No. of manipulated lesions_______

	No. 1	No. 2
localisation		
stage		
excretion of faeces (threads)		
excretion of faeces (liquid)		
expulsion of eggs		
pulsation of the flea		
other observations		

Photo Nos. : ____

Left foot: No. of viable lesions______ No. of manipulated lesions_______

	No. 1	No. 2
localisation		
stage		
excretion of faeces (threads)		
excretion of faeces (liquid)		
expulsion of eggs		
pulsation of the flea		
other observations		

Photo Nos. : ____

CRF-D7: Lesion Viability Scores

Study ID-no.______________ **Day 7** Date_____/_____/ ______Time_______

Right foot: No. of viable lesions______ No. of manipulated lesions_______

	No. 1	No. 2
localisation		
stage		
excretion of faeces (threads)		
excretion of faeces (liquid)		
expulsion of eggs		
pulsation of the flea		
other observations		

Photo Nos. : ____

Left foot: No. of viable lesions______ No. of manipulated lesions_______

	No. 1	No. 2
localisation		
stage		
excretion of faeces (threads)		
excretion of faeces (liquid)		
expulsion of eggs		
pulsation of the flea		
other observations		

Photo Nos. : ____

CRF-E: Lesion Map

Study ID-no.______________ Date_____/______/ 2015

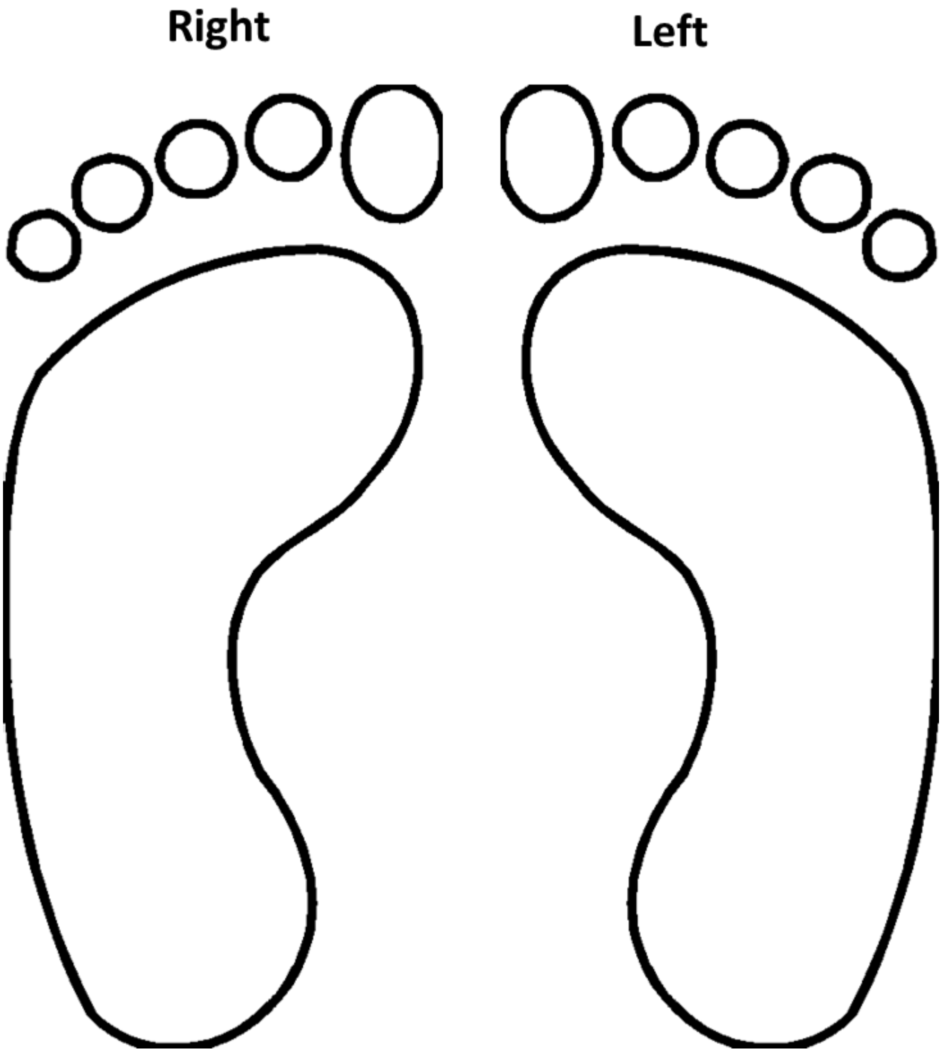

Phase IIa, randomised controlled trial of a locally-made, herbal remedy (neem & coconut oil) for treatment of jiggers (*Tunga penetrans*).

Protocol ID: KEMRI-SERU Non-SSC Protocol No. 514, PPB/ECCT/16/05/03/2016(94)

CRF-F. Adverse Event Log

Patient: ____________________________ **Study: _____________________________**

Study Number: ______________________

AE Term	Grade	Start Date	End Date	Relation to Drug	Action Taken	Intervention Indicated (drug or treatmen

Action Taken

1- None
2- Dose reduced
3- Study drug(s) held/delayed
4- Study drug(s) discontinued
5- Medication given—what kind? (list in intervention column)
6- Hospitalized
7- Other- Specify

Relation to Drug

1. Not related
2. Related to chemotherapy
3. Related to study drug
4. Other- Specify
