# Supplementary material for: Efficacy of a mixture of neem seed oil (Azadirachta indica) and coconut oil (Cocos nucifera) for topical treatment of tungiasis. A randomized controlled, proof-of-principle study
Source: PLoS Negl Trop Dis. 2019 Nov 22;13(11):e0007822. doi: 10.1371/journal.pntd.0007822 (PMC6897421; doi:10.1371/journal.pntd.0007822)
Supplement: S4 Annex — (PDF) [file pntd.0007822.s004.pdf]

Italab Private Limited
INDUSTRIAL TESTING & ANALYTICAL LABORATORIES
Regd. off. : Meher House, 6th Floor, 15, Cawasji Patel Street, Fort, Mumbai - 400 001.
Tel.: 022 - 6780 9988 (10 Lines), 022 - 4333 3888 (10 Lines) • Toll Free : 1800 209 2929
Fax : 022 - 2287 4096, 2801 9800 • E-mail : inquiry@italabs.in • Web : www.italabs.in

CERTIFICATE OF ANALYSIS

Sample Not Drawn By ITALAB

Certificate No. PCD : 015959

Certificate Date : 21/6/2017

1. Particulars of Sample Submitted

Client Name : Elson Consultants
PO Box No. 770,

80202
KENYA

Mfg. Lic. No. : ----

Quantity Received : 100 ml

Client Master No : C009685

Sample Name : Neem Oil

Letter Ref. No. :Email Dt.06.06.2017
Sample Receive Date : 9/6/2017
Marks :--

Manufacturer's Name	Batch/Lot No.	Batch Size/Total Quantity Represented	Date of Mfg.	Date of Exp.
Lynne Elson, Watamu, Kenya	----	----	04/2017	04/2019

2. Result of Analysis :

Parameter Name	Result	Unit
Aflatoxin B1+B2+G1+G2	Not Detected	ppb
Lead (By ICP-OES)	Not Detected	ppm
Mercury (By ICP-OES)	Not Detected	ppm
Zinc (By ICP-OES)	0.15	ppm
Copper (ICP-OES)	0.11	ppm
Chromium (By ICP-OES)	Not Detected	ppm
Cadmium (By ICP-OES)	Not Detected	ppm
**Microbiological Examination**		
Total plate count	10	/ml
Total yeast & mold count	40	/ml
Escherichia coli	Absent	
Salmonella	Absent	

------- END OF REPORT -------

Test Carried Out By :

Vinay 21/06/2017

Mr. Vinay Pujare

For Italab Private Limited

Rathod 21/06/2017

Authorised Signatory

Corp. Off. : Pharmed House, 5th Flr., 141, Walchand Hirachand Marg, Fort, Mumbai - 400 001.
Mktg. Div. : 204, 2nd Floor, Sahyog Bldg., S.V.Road, Kandivali (West), Mumbai - 400 067.
Chennai : Lotus Court, 3rd Floor, New 338, Old 165, Thambu Chetty Street, Chennai – 600 001.
Kolkata : Mercantile Building, 10, Lal Bazaar Street, Kolkata – 700 001.

Collection Centres : Ahmedabad, Bangalore, Hyderabad, New Delhi, Jaipur.

3A® **A Subsidiary of 3A Capital Services Ltd.**

Sr.No.: M037548
