# Supplementary material for: Efficacy of a mixture of neem seed oil (Azadirachta indica) and coconut oil (Cocos nucifera) for topical treatment of tungiasis. A randomized controlled, proof-of-principle study
Source: PLoS Negl Trop Dis. 2019 Nov 22;13(11):e0007822. doi: 10.1371/journal.pntd.0007822 (PMC6897421; doi:10.1371/journal.pntd.0007822)
Supplement: S5 Annex — (PDF) [file pntd.0007822.s005.pdf]

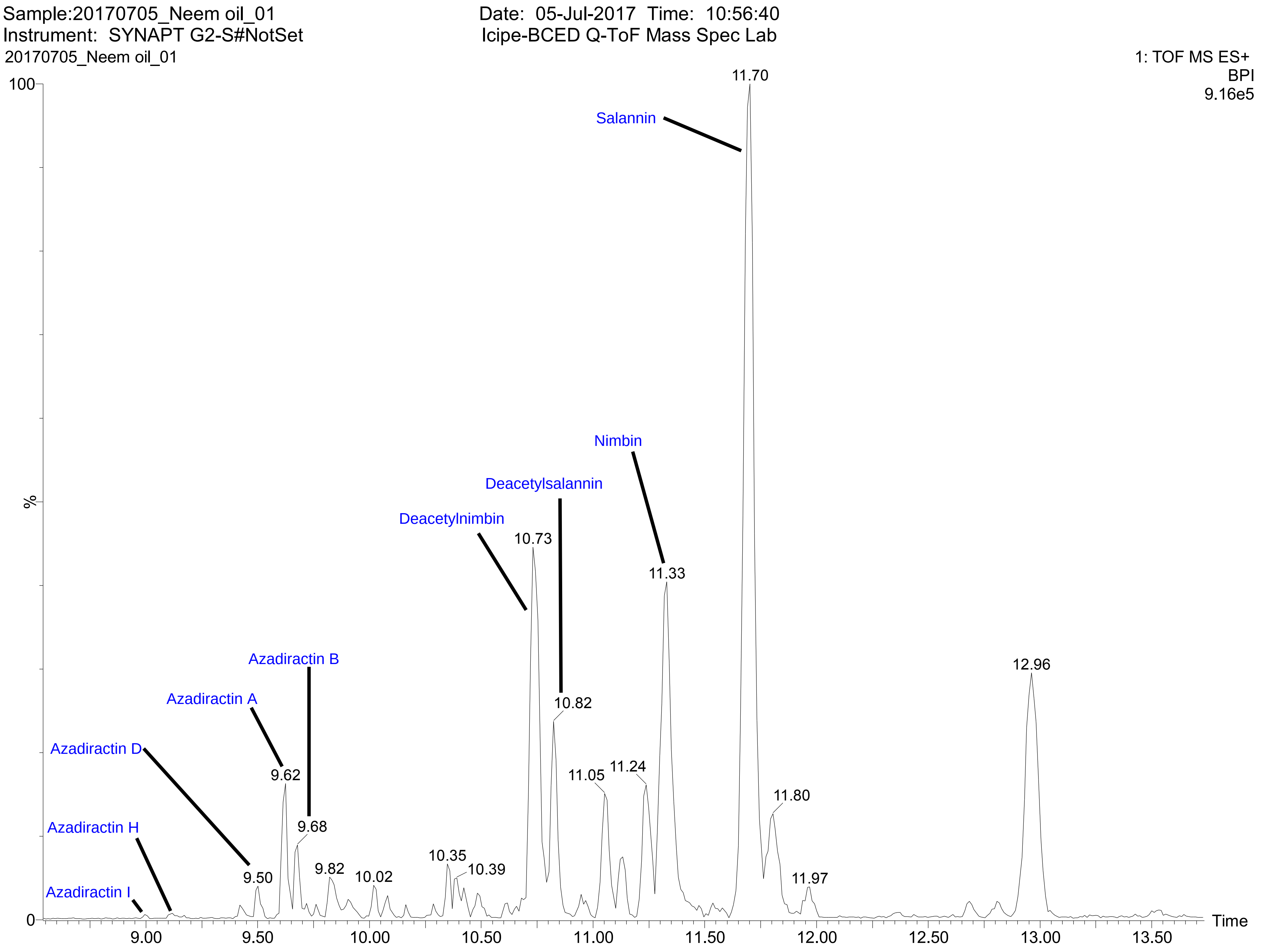

Sample:20170705_Neem oil_01
Instrument: SYNAPT G2-S#NotSet
Date: 05-Jul-2017 Time: 10:56:40
Icipe-BCED Q-ToF Mass Spec Lab
20170705_Neem oil_01
1: TOF MS ES+
BPI
9.16e5
100
%
0
Time
9.00
9.50
10.00
10.50
11.00
11.50
12.00
12.50
13.00
13.50
Salannin
11.70
Nimbin
11.33
Deacetylsalannin
10.82
Deacetylnimbin
10.73
Azadiractin B
9.68
Azadiractin A
9.62
Azadiractin D
9.50
Azadiractin H
Azadiractin I
9.82
10.02
10.35
10.39
11.05
11.24
11.80
11.97
12.96
