# Supplementary material for: Efficacy of a mixture of neem seed oil (Azadirachta indica) and coconut oil (Cocos nucifera) for topical treatment of tungiasis. A randomized controlled, proof-of-principle study
Source: PLoS Negl Trop Dis. 2019 Nov 22;13(11):e0007822. doi: 10.1371/journal.pntd.0007822 (PMC6897421; doi:10.1371/journal.pntd.0007822)
Supplement: S6 Annex — (DOC) [file pntd.0007822.s006.doc]

**1.0 Purpose:**

To determine the chemical composition in neem oil.

**1.1 Experimental**

**1.2.1 Sample preparation**

- 0.03 g of neem oil was dissolved in 1.5 mL Acetonitrile, vortexed for 10 s, ultrasonicated for 30 min, centrifuged at 14 000 rpm for 5 min and the supernatant analyzed by LC-Qtof-MS.
- LC-Qtof-MS analysis the following conditions were employed

| **UPLC** | Waters ACQUITY I-class system |
| --- | --- |
| **UPLC Column** | Waters ACQUITY UPLC BEH C18 column (2.1 × 50 mm, 1.7-μm particle size Waters Corporation, Dublin, Ireland) |
| **Column temperature** | 40°C |
| **Injection volume** | 0.5µl |
| **Mobile phase** | Mobile phases of water (A) and Acetonitrile (B) each with 0.01% formic acid |
| **Flow rate:** | 0.3 ml/min, Gradient flow mode (start 95% A to 100 %B back to starting gradient. Run time 25 min |
| **Q-tof conditions** |  |
| **Ion mode** | Both positive and negative mode (for the reporting in this analysis only result for positive are provided) |
| **nitrogen desolvation flow rate** | 500 l/h |
| **accuracy** | < 5ppm |

**2.0 Results**

**Table 1:** Retention times, peaks in ESI positive mass spectra and attribution for neem oil

| **RT**  **(min)** | **Compound name** | **ESI + ve (m/z)** | **Attribution** |
| --- | --- | --- | --- |
| 9.01 | Azadiractin I | 641.2571 | [M+Na]+ |
|  |  | 619.2786 | [M+H]+ |
| 9.16 | Azadiractin H | 685.2477 | [M+Na]+ |
|  |  | 663.2725 | [M+H]+ |
|  |  | 645.2383 | [M+H-H2O]+ |
|  |  | 627.244 | [M+H-2H2O]+ |
| 9.50 | Azadiractin D | 699.2634 | [M+Na]+ |
|  |  | 677.2448 | [M+H]+ |
|  |  | 659.2714 | [M+H-H2O]+ |
| 9.62 | Azadiractin A | 743.2523 | [M+Na]+ |
|  |  | 721.2896 | [M+H]+ |
|  |  | 703.2607 | [M+H-H2O]+ |
|  |  | 685.2501 | [M+H-2H2O]+ |
| 9.68 | Azadiractin B | 685.2474 | [M+Na]+ |
|  |  | 663.2741 | [M+H]+ |
|  |  | 645.2551 | [M+H-H2O]+ |
|  |  | 627.2443 | [M+H-2H2O]+ |
| 10.73 | Deacetylnimbin | 521.2161 | [M+Na]+ |
|  |  | 499.2353 | [M+H]+ |
| 10.82 | Deacetylslannin | 577.2833 | [M+Na]+ |
|  |  | 555.2988 | [M+H]+ |
| 11.33 | Nimbin | 563.2266 | [M+Na]+ |
|  |  | 541.2485 | [M+H]+ |
| 11.70 | Slannin | 619.2889 | [M+Na]+ |
|  |  | 597.3184 | [M+H]+ |

**Reference:**

Barrek, S., Paisse, O., & Grenier-Loustalot, M. F. (2004). Analysis of neem oils by LC-MS and degradation kinetics of azadirachtin-A in a controlled environment : Characterization of degradation products by HPLC-MS-MS*. Analytical and Bioanalytical Chemist*ry*, 3*78(3), 753–763. doi:10.1007/s00216-003-2377-0

Beltrán, E., Ibáñez, M., Gracia-Lor, E., Sancho, J. V., Hernández, F., & Thompson, D. G. (2014). Application of liquid chromatography/mass spectrometry in assessment of potential use of azadirachtins (TreeAzin ^TM^ ) against Asian longhorned beetle. *Anal. Methods*, *6*(19), 8063–8071. doi:10.1039/C4AY01553C
